# Environmental Drivers of *Bacillus*-Positive Blood Cultures in a Cancer Hospital, Sapporo, Japan

**DOI:** 10.3390/ijerph15102201

**Published:** 2018-10-09

**Authors:** Takahiro Fujita, Hiroshi Nishiura

**Affiliations:** 1 Hokkaido Cancer Center, Kikusui, Sapporo 003-0804, Japan; tfujita@sap-cc.go.jp; 2 Graduate School of Medicine, Hokkaido University, Kitaku, Sapporo 060-8638, Japan

**Keywords:** disease outbreak, environmental health, epidemiologic studies, nosocomial infections, hospitals

## Abstract

The *Bacillus* species is a well-documented causative pathogen of nosocomial bloodstream infection. The present study aimed to identify climatological variables that are associated with *Bacillus*-positive blood culture in Sapporo, Japan. All cases with *Bacillus*-positive blood cultures from January 2011 to December 2016 were retrospectively analysed. Climatological data from 2011 to 2016, including daily mean temperature and absolute humidity, were retrieved from the Japan Meteorological Agency. Employing a hazard-based statistical model to describe the non-homogeneous counting process in which temperature and absolute humidity act as explanatory variables, we computed all possible models with variable lengths of time lag. Akaike Information Criterion was computed to identify the best fitted model. High wavelet power at 12 months was identified for the period from 2013 onwards, which coincided with the time period in which sampling multiple sets of blood culture has been recommended. The temperature-only model with a lag of six days yielded a high sensitivity value (72.1%) and appeared to be the optimal model to predict *Bacillus*-positive blood culture with the highest area under the receiver operating characteristic curve value. Temperature was identified as a climatological driver of *Bacillus*-positive blood culture. Our statistical modelling exercise offers an important message for infection control practices to improve awareness among healthcare workers of the identified association and mechanically controlled in-room temperature.

## 1. Introduction

Bacteria of the genus *Bacillus*, which are ubiquitous in the environment, are Gram-positive, rod-shaped spore-forming pathogens. *Bacillus* spp. optimally grow at temperatures ranging from 25 to 37 °C on standard culture medium [1]. *Bacillus* spp., including highly abundant *Bacillus cereus*, are well-documented pathogens of nosocomial bloodstream infection (BSI) in immunocompromised patients and patients with malignancies [2]. Adherence of some *Bacillus* spp. to catheters is considered to frequently induce catheter-related BSI [3]. *Bacillus* spp. are also considered as a contaminant when isolated from clinical specimens, including blood culture. Hospital outbreaks of *Bacillus* spp. in a variety of settings such as the intensive care unit (ICU) [4], neonatal ICU [5], and surgical patients [6] have been reported. Moreover, pseudo-outbreaks induced by the contamination of hospital environments have been reported [7,8].

In Japan, *B. cereus* bacteraemia outbreaks due to contaminated linen and reusable towels have been reported. Many of these outbreaks occurred in summer [9,10]. Furthermore, the recovery of *Bacillus* spp. from blood culture is known to peak in summer in Japan [11,12]. Seasonality of *Bacillus* spp. isolated from blood culture has also been implicated [13]. Nevertheless, environmental drivers of *Bacillus* spp. have not been explored in detail, especially with the aim of predicting the risk of positive blood cultures using environmental data.

The abovementioned published evidence endorses the concept that the recovery of *Bacillus* spp. may be closely associated with climatological determinants. Whereas a small number of published studies have emphasized that high temperature is likely associated with *Bacillus* spp. [10,11], identification of the exact climatological predictors and formulation of predictive models are called for. Therefore, the purpose of the present study was to epidemiologically identify climatological variables that are associated with *Bacillus*-positive blood culture in Sapporo, Japan, by employing a hazard-based statistical model.

## 2. Materials and Methods

### 2.1. Blood Culture Sampling

All cases with *Bacillus*-positive blood culture from January 2011 to December 2016 were retrospectively analysed using the laboratory database of microbiological testing at Hokkaido Cancer Center, a 520-bed tertiary cancer hospital in Sapporo, Japan. Blood culture samples were obtained from patients with suspected bacteraemia according to physicians’ decision. The date on which sampling occurred for each *Bacillus*-positive blood culture was examined, regardless of the actual bacteraemia or contamination of the sample. Days without positive patients were also recorded. All other datasets were anonymised and unused. Because the present study aimed to identify environmental drivers of *Bacillus* spp., which are rare events and hereafter modelled the occurrence as the stochastic counting process over time, we discarded the information on individual patients’ characteristics. All admitted patients in the corresponding hospital have neoplasms for treatment, and the underlying characteristics of admitted patients do not greatly vary with time.

### 2.2. Microbiological Method

Blood culture specimens were inoculated into BacT/ALERT SA and SN bottles in a BacT/ALERT 3D system (bioMérieux, Marcy l’Etoile, France). Species identification was performed with BBL Crystal (Becton Dickinson, Sparks, MD, USA) Gram-positive identification panels. Our data set is the binary data describing the daily occurrence of *Bacillus*-positive blood culture (i.e., comparing *Bacillus* positive dates against no *Bacillus* dates), and the measurement unit was per day, allowing an identification of the day with a high risk of infection.

### 2.3. Environmental Variables

Climatological data from 2011 to 2016, including daily mean temperature, mean relative humidity, total hours of sunlight per day, and wind speed, were retrieved from the website of the Japan Meteorological Agency (http://www.jma.go.jp/jma/indexe.html). All datasets were collected at the observatory weather station in Sapporo. Sapporo is located approximately at latitude 43°4′ and longitude 141°21′ and has a humid continental climate. The average daily mean temperature is around 22 °C in summer (August) and around −3 °C in winter (February). Temperature, relative humidity, and wind speed were provided as the daily average in each geographic observation point. Absolute humidity was calculated using temperature and relative humidity. Collected environmental data are available as Supplementary Table S1.

### 2.4. Statistical Analysis

First, cyclical behaviour of the incidence of *Bacillus*-positive blood culture was examined by employing wavelet analysis because a causal link to environmental drivers would be plausible when the incidence also varies with seasonality. The wavelet power spectral density, i.e., a low-resolution equivalent to the Fourier transform, was employed. The wavelet power is equivalent to the amplitude-squared, and identifying regions of large power within the power spectrum helps determine which features of signal are important and which can be ignored. If the behaviour was not cyclical and a consistent sampling scheme was not employed over time, we removed these sampling periods and further examined only the data with an apparent signature of seasonality in the following analyses.

Second, we conducted univariate analyses to examine the association between climatological variables and *Bacillus*-positive blood culture by employing a logistic regression method. The logistic regression method was adopted for the convenient calculation of the odds ratio, and moreover, the use of the method for the *Bacillus*-positive blood culture was mathematically supported from a practical point of view, because the infection has been known to be of environmental origin (e.g., contaminated warm towel) [9,10] and dependence between the events can be ignored. Mean temperature (°C), absolute humidity (g/m^3^), hours of sunlight, and wind speed (m/s) were used as climatological variables. This process enabled us to reject a model with variables that are not associated with *Bacillus*-positive blood culture in the subsequent analyses.

Third, we analysed the risk of observing *Bacillus*-positive blood culture using a hazard-based model. Specifically, we employed a non-homogeneous counting process model to describe the temporal event frequency of *Bacillus*-positive blood culture, modelling the daily risk. For this infection of environmental origin, we assume that the underlying characteristics of inpatients were unchanged over time, and model the daily risk using the time-dependent hazard that is governed by environmental variables. Let *λ_t_* be the hazard of a positive outcome at calendar time *t*. To parameterize the hazard function, we used variables that appeared to be predictive in univariate analyses. Lag days of climatological variables were set to account for the background causal mechanism of environmental variables influencing the risk of *Bacillus* spp. The daily hazard function reads as follows:

(1) $$\;\lambda_{t}=\exp\left(a_{0}+\underset{i}{\sum }a_{i}x_{i}\left(t\right)\right)\;$$

where *a*_0_ represents the baseline, *a_i_* is the coefficient of variable *i*, and *x_i_*(*t*) is the input variable *i* that varies as a function of day *t*. The model was dealt with as a multivariate model. When we used multiple input variables *x_i_*, we also accounted for an additional term (e.g., *x*_1_*x*_2_) to address interaction between two variables. Let *U* and *W* be the groups of days with and without *Bacillus*-positive blood cultures, respectively. The likelihood function to estimate parameters of the hazard (1) is

(2) $$\;L\left(\theta ;D\right)=\underset{t_{k}\in U}{\prod }\lambda_{t_{k}}\left(\theta \right)\underset{t_{k}\in W}{\prod }\left(1-\lambda_{t_{k}}\left(\theta \right)\right)\;$$

To obtain maximum likelihood estimates, the negative logarithm of Equation (2) was minimized.

To compare different models (e.g., models with different variables or models with different lengths of time lag), Akaike Information Criterion (AIC) was used, reflecting a penalty in the number of parameters. The diagnostic performance of the prediction model, including sensitivity, specificity, and area under the receiver operating characteristic curve (AUC), was then quantified along with the positive predictive value (PPV) and negative predictive value (NPV). To identify the optimal threshold of the predicted risk, we used the Youden index (i.e., sensitivity plus specificity minus 1). The 95% confidence intervals (CI) of the sensitivity and specificity were computed using normal approximation to binomial distribution, while calculations of 95% CIs of the PPV and NPV, as well as the AUC, were based on the Wald method as described elsewhere [14]. As our subject sample size was small, the predictive assessment is subject to overoptimism. That is, our model might fit the data which have been used to estimate the parameters better than it fits new data. To avoid overly optimistic results, we implemented sequential evaluations. Namely, we predicted 2015–2016 and 2016 data using training datasets up to 2014 and 2015, respectively.

All statistical analyses were conducted using JMP Pro 13 (SAS Institute Inc., Cary, NC, USA) and statistical package R (https://cran.r-project.org/) [15].

### 2.5. Ethical Consideration

The present study only analysed the counts of data collected in a hospital, and the data were fully anonymised in advance of our analysis. Moreover, the climatological datasets are publicly available. Thus, we did not require patient consent. The Medical Ethics Committees at the Graduate School of Medicine, Hokkaido University and the Hokkaido Cancer Center approved this study.

## 3. Results

### 3.1. Descriptive Data

**Figure 1:**
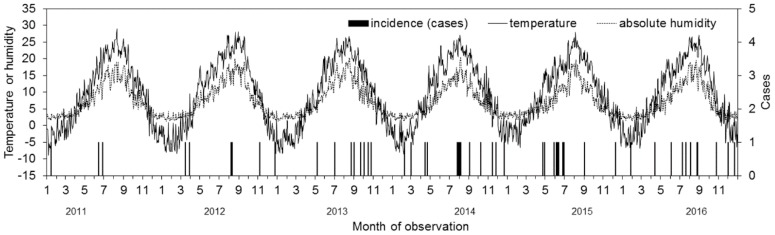
Comparison between climatological variables and frequency of blood culture positives with *Bacillus* spp. from 2011–2016. Daily average temperature and absolute humidity (g/m^3^) in Sapporo city were retrieved from a local weather station. Incidence of positive blood culture with *Bacillus* spp. are measured on the right vertical axis. Date of incidence on the horizontal axis is measured by the date of sample collection.

From 2011 to 2016, a total of 52 blood samples tested positive for *Bacillus* spp. Of these, 42 (80.7%) were *B. cereus*, 4 (7.7%) were *B. subtilis*, 2 (3.8%) were *B. megaterium*, and 4 (7.7%) were reported as *Bacillus* spp. (i.e., unidentified). The mean and standard deviation of the age were 62.9 and 14.7 years, respectively. Twenty-three (44.2%) were male. Figure 1 visually presents the occurrence of *Bacillus* spp. in contrast to the daily temperature, as well as the absolute humidity. Overall, 26 (50.0%) *Bacillus*-positive blood cultures occurred from June to September, i.e., during the summer season. The two clinical wards that patients were most frequently admitted to were gastroenterology and urology (*n* = 10 and 10, respectively). Individual patients’ background data are available as Supplementary Table S2.

**Figure 2:**
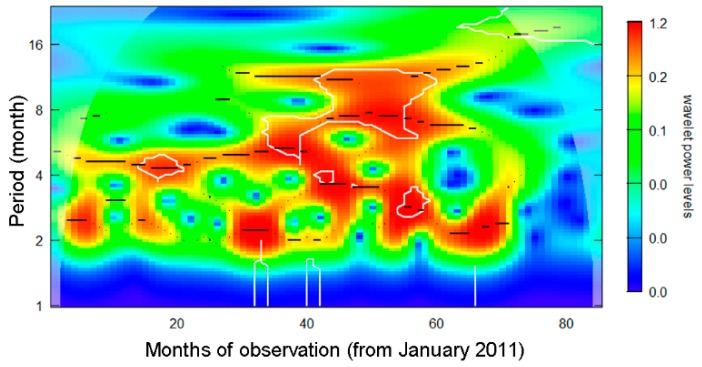
Wavelet power spectrum analysis of blood culture positives with Bacillus spp from 2011–2016. Horizontal axis counts month since January 2011. High power density indicates possible periodic occurrence of blood culture positives. For instance, red hot spot at 12-month period from 2013–2016 supports annual cyclical behavior during the corresponding period.

Figure 2 shows the result of wavelet analysis. High wavelet power at 12 months was identified for the period from 2013 onwards. Thus, blood culture data from 2011–2012 were omitted from subsequent analyses. Such removals would be practically supported, because *Bacillus*-positive blood cultures are identified by ensuring routine testing with a multiple set culture, and in fact, the very high frequency of single set culture was seen during the period from 2011–2012 (ranging from 75 to 90%). Recommendation for sampling multiple sets of blood for culture was issued in 2013, and the recent multiple sampling rate was as high as 92.0%.

### 3.2. Univariate Analysis

Table 1 shows the univariate association between the incidence of Bacillus-positive blood culture and climatological variables. High mean temperature and absolute humidity appeared as positive predictors of incidence, with estimated odds ratios of 1.04 (95% CI: 1.01, 1.08) and 1.07 (95% CI: 1.00, 1.13), respectively. That is, a warm and wet environment likely promoted *Bacillus* spp.; however, the univariate association could still reflect them as seasonal confounders. Including 2011–2012 data which did not frequently implement multiple set sampling, temperature was still a weak predictor (*p* = 0.05), but humidity was not significantly associated with the occurrence of *Bacillus*-positive culture (*p* = 0.74).

### 3.3. Multivariate Analysis

**Figure 3:**
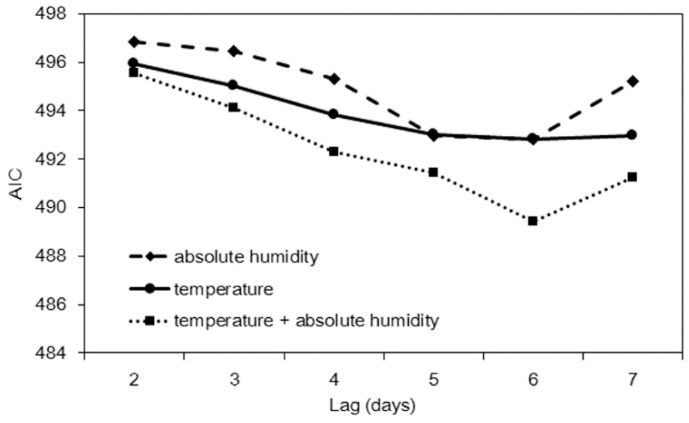
Penalized likelihood, Akaike Information Criterion (AIC), for three prediction models with different time lags. All three models were best fitted to the data with a time lag of six days.

Using temperature and/or absolute humidity with variable time lags, we computed all possible models and compared AIC values. Figure 3 shows the result of model comparison. Setting a six-day lag appeared to yield the minimum AIC value for all three models, and the use of both temperature and absolute humidity appeared to yield the minimum AIC value. Model performance is compared in Table 2. The temperature-only model and humidity-only model yielded high sensitivity values (72.1%), while the highest specificity value was observed with the combination of the two variables (56.0%). Concerning predictive performance, the temperature-only model appeared to yield the highest AUC value while using only one input variable.

**Figure 4:**
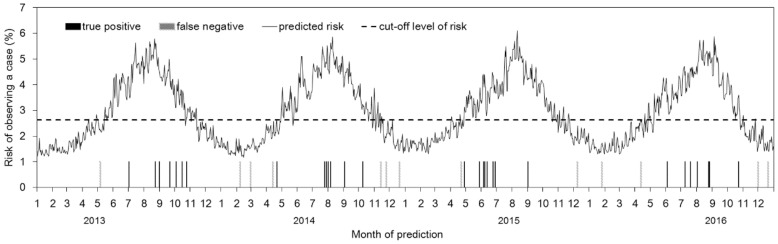
Predicted risk of observing a positive blood culture of *Bacillus* spp. Employing the temperature alone with a lag of six days, the risk of observing a case on each given day was estimated. Black bars represent true positive events of positive blood culture, whereas gray bars represent false negative events.

**Figure 5:**
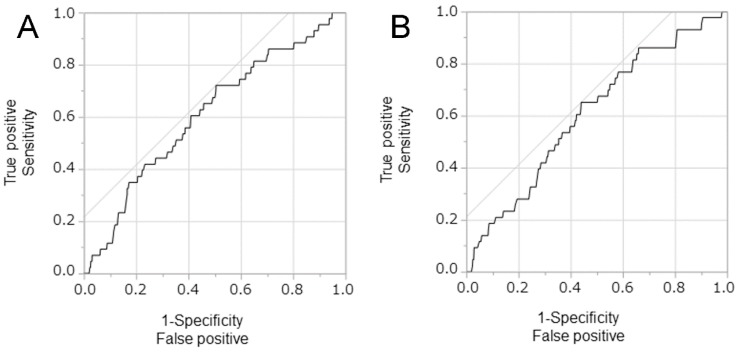
Receiver Operator Characteristic curves of a positive blood culture of *Bacillus* spp. (**A**) Prediction using the temperature alone and (**B**) prediction based on temperature plus absolute humidity. The relationships between sensitivity (true positives) and 1-specificity (true negatives) in determining the diagnostic performances of these climatological variables for predicting a positive blood culture of *Bacillus* spp. In both panels, the time lag of six days was taken.

Using temperature only, Figure 4 visually shows how our model performs in predicting the incidence of *Bacillus* spp. Of a total of 43 events of *Bacillus*-positive blood cultures, 30 events (69.8%) were successfully predicted by our environment-based prediction model. Figure 5 compares the receiver operating characteristic (ROC) curves of the two best-fit models, i.e., temperature-only and temperature plus absolute humidity. Both models appeared as comparable and weak prediction models, i.e., while improving incidence prediction, the predictive performance is limited and the ROC curve is distant from the upper left corner.

Figure 5 compares the receiver operating characteristic (ROC) curves of the two best-fit models, i.e., temperature-only and temperature plus absolute humidity. Both models appeared as comparable and weak prediction models, i.e., while improving incidence prediction, the predictive performance is limited and the ROC curve is distant from the upper left corner.

To avoid overly optimistic results, we predicted 2015–2016 and 2016 data using the training data up to the end of 2014 and 2015, respectively. Out of the total of 22 and 12 events with *Bacillus*-positive samples in 2015–2016 and 2016, a total of three and eight events were successfully predicted. Thus, success rates were 13.6% (95% CI: 0, 27.3) and 66.7% (95% CI: 41.7, 91.7), respectively, in 2015–2016 and 2016.

## 4. Discussion

The present study explored the predicted risk of detecting *Bacillus* spp. from blood culture using the hazard-based counting process model. The dataset was derived from a designated cancer hospital in Sapporo, Japan, in which sampling multiple sets of blood culture has been issued since 2013 and an annual cyclical pattern of the incidence of *Bacillus*-positive blood culture has been observed. To model *Bacillus*-positive blood culture, we explored the predictive performance of climatological variables, including temperature and absolute humidity. Based on model comparison using penalized likelihood, we have shown that the mean temperature with a time lag of six days would optimally predict the incidence of *Bacillus* spp. from blood culture, irrespective of true bacteraemia or contamination.

To our knowledge, the present study is the first to explicitly identify a climatological driver of *Bacillus*-positive blood culture. A published study indicated the presence of seasonality in the frequency of *Bacillus*-positive blood culture and identified a possible association with high temperature [13]. While temperature and humidity were considered as potential environmental determinants, the contribution of the present study is that we rigorously identified that temperature with a certain time lag (six days) would be predictive of the incidence employing a hazard-based statistical model. Although the predictive performance was overall limited, we have shown that temperature is a sensitive epidemiological determinant of the incidence of *Bacillus* spp. While the environmental driver was identified, the predictive performance was limited with the calculated AUC at around 0.6. Moreover, when we sequentially predicted the incidence in 2015–2016 and 2016 using the rest of time as the training data, the success rate appeared to be very small. Thus, the predictive performance of the resulting model was weak, and the biggest contribution was the identification of temperature itself.

The most common clinical presentation of *Bacillus* bacteraemia is catheter-related BSI. Thus, risk factors of *Bacillus* bacteraemia that have been investigated include the presence of a central venous catheter [16], prior use of extended-spectrum cephalosporin [17], and usage of peripheral parenteral nutrition solutions [18]. Because exposure to these risk factors is unlikely to be seasonal, we considered that environmental exposure to *Bacillus* spp. in patient rooms would act as the most probable driver of *Bacillus*-positive blood culture. In fact, *Bacillus* outbreaks that were associated with the contamination of reusable towels and linens have been reported [10,19]. While published studies have also indicated that *Bacillus* outbreaks were associated with hospital reconstruction or renovation activities [7,20,21,22], no construction took place during our study period at our subject hospital; thus, our identification of an epidemiological association between *Bacillus* spp. and environmental predictors was not caused by coincidence of construction.

Our findings involve important clinical implications. First, awareness of the association between *Bacillus*-positive blood cultures and temperature would be crucial for preventing bacteraemia, as well as blood culture contamination. As part of infection control activities, healthcare workers should be informed of our finding to be aware of the elevated risk of infection during and following warm days. Second, the association between temperature and *Bacillus*-positive blood culture would also imply the importance of controlling temperature at caregiving settings, e.g., the use of air conditioning systems during the summer season.

The present study has some limitations. First, this study was conducted in a retrospective manner with a relatively small sample size. As such, the level of evidence is limited. Moreover, sampling error cannot be immediately overcome. Nevertheless, our finding of the seasonality and identification of temperature associated with the incidence of *Bacillus*-positive blood culture was consistent with published studies, and we believe that the plausibility is well supported. Second, our study relied on observation at a single healthcare centre; thus, representativeness may be limited and our findings may not be directly applicable to all other settings. Third, environmental variables were observed at an outer environment in Sapporo. However, ordinary clinical wards of our subject cancer hospital, built in the 1970s, have not been equipped with air conditioning facilities (i.e., cooling system was absent) and windows are continuously opened to lower in-room temperature during the summer season.

In conclusion, we have shown that *Bacillus*-positive blood cultures exhibit an annual cyclical pattern with high incidence during the summer season, and the incidence was weakly but validly predicted by mean temperature in Sapporo. We believe that our statistical modelling exercise offers an important clue for infection control practice to improve awareness among healthcare workers of the identified association and mechanically controlled in-room temperature.

## 5. Conclusions

The present study aimed to epidemiologically identify climatological variables that are associated with *Bacillus*-positive blood culture in Sapporo, Japan, by employing a hazard-based statistical model. Using temperature and/or absolute humidity with variable time lags, we computed all possible models. Based on model comparisons, the temperature-only model with a lag of six days yielded a high sensitivity value (72.1%) and appeared to be the optimal model to predict *Bacillus* spp. with the highest AUC value. Our statistical modelling exercise offers an important message for infection control practice to improve awareness among healthcare workers of the identified association and mechanically controlled in-room temperature.

## Figures and Tables

**Table 1:** Univariate association between the incidence of *Bacillus* spp. and climatological variables.

Variables	Odds Ratio (95% CI ^†^)	*p*-Value
Mean temperature (Celsius)	1.04 (1.01, 1.08)	0.018
Absolute humidity (g/m^3^)	1.07 (1.00, 1.13)	0.033
Hours of sunlight (hours)	1.02 (0.94, 1.10)	0.621
Speed of wind (m/s)	0.94 (0.75, 1.13)	0.516

^†^ CI, confidence interval.

**Table 2:** Estimated diagnostic performance of climatological prediction models for forecasting the incidence of *Bacillus* spp. blood culture.

Variable (Lag Days)	Sensitivity	Specificity	PPV	NPV	AUC	AIC
Mean temperature (Celsius) (6 days)	72.1 (56.3, 84.7)	49.5 (46.8, 52.1)	4.1 (2.8, 5.8)	98.3 (97.1, 99.1)	0.61 (0.59, 0.64)	492.8
Absolute humidity (g/m^3^) (6 days)	72.1 (56.3, 84.7)	48.8 (46.1, 51.4)	4.1 (2.8 5.8)	98.3 (97.0 99.1)	0.59 (0.57, 0.62)	492.8
Mean temperature + Absolute humidity (6 days)	65.1 (49.1, 79.0)	56.0 (53.3, 58.6)	4.3 (2.9, 6.1)	98.1 (97.0, 99.0)	0.61 (0.58, 0.63)	489.4

PPV: Positive predictive value; NPV: Negative predictive value; AUC: Area under the curve; AIC: Akaike Information Criterion. Sensitivity, specificity, PPV, and NPV are expressed as percentages. Values in parentheses represent the 95% confidence interval.

## Supplementary Materials

The following are available online at http://www.mdpi.com/1660-4601/15/10/2201/s1, Table S1: Climatological variables and incidence in Sapporo from 2013–16, Table S2: Background demographic characteristics, date of sampling and admitted ward of cases.
