# Supplementary material for: Environmental Drivers of Bacillus-Positive Blood Cultures in a Cancer Hospital, Sapporo, Japan
Source: Int J Environ Res Public Health. 2018 Oct 9;15(10):2201. doi: 10.3390/ijerph15102201 (PMC6210759; doi:10.3390/ijerph15102201)
Supplement: Supplementary file 1 [file ijerph-15-02201-s001.pdf]

Table S1. Climatological variables and incidence in Sapporo from 2013–16.

Date	Mean_temperature (Celsius)	Mean_relative _humidity (%)	Absolute_humidity (g/m^3)	Mean_speed _of_wind (m/s)	Incidence
2013/1/1	-5.6	64	2.084420238	2	0
2013/1/2	-4.3	67	2.396800515	3.1	0
2013/1/3	-6.4	42	1.290451857	5.2	0
2013/1/4	-7.6	54	1.519066681	4.1	0
2013/1/5	-4.2	58	2.089779452	3.1	0
2013/1/6	-5.9	67	2.135054284	4.2	0
2013/1/7	-7.1	58	1.692868735	3.4	0
2013/1/8	-7.3	54	1.55307619	2.8	0
2013/1/9	-8.2	61	1.641358042	1.9	0
2013/1/10	-6.6	52	1.57448828	3.1	0
2013/1/11	-6.5	59	1.799565801	3.3	0
2013/1/12	-4.5	71	2.503666173	2.3	0
2013/1/13	-3.9	57	2.098345277	4.5	0
2013/1/14	-5.3	64	2.13025337	1.6	0
2013/1/15	-7.1	74	2.159867007	3.2	0
2013/1/16	-6.6	70	2.119503454	1.5	0
2013/1/17	-8.3	73	1.949700784	1.6	0
2013/1/18	-8	74	2.02093941	3.3	0
2013/1/19	-2.4	75	3.071450993	5.1	0
2013/1/20	-1.1	84	3.768614141	5.8	0
2013/1/21	-1.1	79	3.544291871	3.2	0
2013/1/22	-2.7	69	2.76645821	1.4	0
2013/1/23	-3.9	75	2.760980627	1.3	0
2013/1/24	-2.6	72	2.90722948	4.3	0
2013/1/25	-1	67	3.026957298	4.9	0
2013/1/26	-4.9	59	2.021430879	3.2	0
2013/1/27	-2.3	77	3.175680298	6.8	0
2013/1/28	-4.3	72	2.575666226	1.5	0
2013/1/29	-4.5	78	2.7505065	2.8	0
2013/1/30	-1	79	3.569098904	5.8	0
2013/1/31	-0.8	79	3.619169118	1.5	0
2013/2/1	1.7	72	3.91798871	4.4	0
2013/2/2	1.2	64	3.365813153	5.9	0
2013/2/3	-5.8	62	1.990149392	5.2	0
2013/2/4	-3.1	72	2.806036622	2.4	0
2013/2/5	-4.9	56	1.918646258	6.1	0
2013/2/6	-4.1	61	2.2136804	1.3	0
2013/2/7	-3.5	77	2.916715007	4.4	0
2013/2/8	-2.5	78	3.17183451	6.8	0
2013/2/9	-4.7	73	2.537411332	4.4	0
2013/2/10	-5.8	70	2.246942861	1.7	0
2013/2/11	-6.1	71	2.229791668	1.6	0
2013/2/12	-6	72	2.277738871	1.9	0
2013/2/13	-3.8	70	2.595401375	1.8	0

2013/2/14	-2.4	82	3.358119753	1.3	0
2013/2/15	-3.3	61	2.343782604	3	0
2013/2/16	-5.7	77	2.489669938	2.8	0
2013/2/17	-5.9	69	2.198787248	3.2	0
2013/2/18	-5.3	82	2.729387131	2	0
2013/2/19	-4	75	2.74129785	2.3	0
2013/2/20	-6.4	58	1.782052564	3.7	0
2013/2/21	-5.4	59	1.9496535	4.1	0
2013/2/22	-5	62	2.108938166	2.3	0
2013/2/23	-5.3	58	1.930542117	3.3	0
2013/2/24	-6.9	77	2.280714262	2	0
2013/2/25	-5.8	71	2.279042045	2.5	0
2013/2/26	-3.4	54	2.060110436	2.8	0
2013/2/27	-0.3	65	3.083009567	4.6	0
2013/2/28	0.8	69	3.530664303	3.7	0
2013/3/1	0.6	79	3.987196311	5.9	0
2013/3/2	-0.8	78	3.573356851	9	0
2013/3/3	-2.6	73	2.947607667	6.7	0
2013/3/4	-2.7	77	3.087206988	2	0
2013/3/5	-0.6	66	3.065952958	1.7	0
2013/3/6	0.3	71	3.510178992	4.2	0
2013/3/7	0.6	76	3.835783793	5.1	0
2013/3/8	1.4	76	4.051907839	3.3	0
2013/3/9	-5.4	72	2.379238169	6.7	0
2013/3/10	-4	69	2.521994022	6.2	0
2013/3/11	-3.7	61	2.277917461	4	0
2013/3/12	2.7	65	3.78524952	2.8	0
2013/3/13	2	84	4.665215808	4.5	0
2013/3/14	-3.2	56	2.167023143	4.6	0
2013/3/15	1.5	59	3.167102403	3.4	0
2013/3/16	-0.8	72	3.298483247	4.9	0
2013/3/17	0.8	58	2.967804777	4.2	0
2013/3/18	4.8	76	5.093601249	4.8	0
2013/3/19	3.4	64	3.906847518	3.6	0
2013/3/20	-0.4	74	3.485640026	3.9	0
2013/3/21	-4	66	2.412342108	5.5	0
2013/3/22	-0.1	70	3.366457338	4.1	0
2013/3/23	-1.7	55	2.366109507	3.5	0
2013/3/24	0.1	56	2.730651181	2.4	0
2013/3/25	-0.1	58	2.789350366	1.9	0
2013/3/26	0.4	66	3.285544184	3.3	0
2013/3/27	2.8	54	3.165964662	5.3	0
2013/3/28	4.6	80	5.290978318	4	0
2013/3/29	2.4	60	3.423921878	2.4	0
2013/3/30	1	60	3.11254098	2.8	0
2013/3/31	1.3	52	2.753484416	2.3	0
2013/4/1	3.2	48	2.89103276	3.2	0
2013/4/2	5.2	64	4.404459646	2.7	0
2013/4/3	6.9	74	5.693662883	3.8	0
2013/4/4	5.9	56	4.035842022	4	0

2013/4/5	4.6	58	3.83595928	2.1	0
2013/4/6	7.4	65	5.166430185	6.7	0
2013/4/7	9.3	90	8.084009038	5.4	0
2013/4/8	5.3	61	4.225827026	7.9	0
2013/4/9	5.4	59	4.114344717	5.5	0
2013/4/10	4.8	59	3.954243075	3.3	0
2013/4/11	4.5	60	3.94194791	3.1	0
2013/4/12	3.1	71	4.247652863	2.7	0
2013/4/13	6.4	52	3.872455818	2.8	0
2013/4/14	8.3	68	5.728589682	7.6	0
2013/4/15	5.7	46	3.271841476	3.5	0
2013/4/16	6.7	51	3.873170653	3.4	0
2013/4/17	4.4	81	5.286348908	4.1	0
2013/4/18	4.9	71	4.790145421	3.5	0
2013/4/19	3.4	76	4.639381428	4.3	0
2013/4/20	5.5	57	4.00117797	3	0
2013/4/21	7.1	49	3.819531774	4	0
2013/4/22	8.6	45	3.864821874	4.4	0
2013/4/23	10.7	45	4.417651677	2.6	0
2013/4/24	9.7	59	5.436374744	5.7	0
2013/4/25	8.8	64	5.567634085	4.4	0
2013/4/26	8.6	72	6.183714998	4.3	0
2013/4/27	7	83	6.427858735	6.3	0
2013/4/28	5.4	81	5.648507154	10.4	0
2013/4/29	5.8	80	5.727715977	6.2	0
2013/4/30	5.1	81	5.537656592	4.1	0
2013/5/1	5.5	79	5.545492274	2.3	0
2013/5/2	4.6	71	4.695743257	6.5	0
2013/5/3	4.6	84	5.555527234	3.8	0
2013/5/4	5.9	86	6.197900249	2.1	0
2013/5/5	7.8	79	6.443996415	1.7	0
2013/5/6	6	81	5.876021481	3.2	1
2013/5/7	5	56	3.803250451	6.2	0
2013/5/8	7.8	55	4.486326618	5.8	0
2013/5/9	11.2	61	6.179935127	2.9	0
2013/5/10	12.9	57	6.421000382	4.6	0
2013/5/11	9.8	77	7.140203614	8.1	0
2013/5/12	10.7	79	7.755432943	2.8	0
2013/5/13	8.7	76	6.569292513	6	0
2013/5/14	10.3	79	7.561795499	2.6	0
2013/5/15	9.6	80	7.324577136	4.4	0
2013/5/16	9.7	84	7.739923365	4.2	0
2013/5/17	12.1	69	7.395641968	2.6	0
2013/5/18	12.8	70	7.836705839	2.1	0
2013/5/19	12.8	67	7.500847017	3.1	0
2013/5/20	12.7	78	8.678317818	4.5	0
2013/5/21	14.7	69	8.682844066	3	0
2013/5/22	13	81	9.181274547	2.6	0
2013/5/23	9.7	90	8.292775034	4.6	0
2013/5/24	10	74	6.949757846	4.7	0

2013/5/25	13.3	66	7.62112257	2.7	0
2013/5/26	16.4	61	8.509616855	4.8	0
2013/5/27	19.6	54	9.111357458	2.8	0
2013/5/28	20	60	10.36376522	5.6	0
2013/5/29	15.3	78	10.18072575	7.6	0
2013/5/30	16.9	71	10.20675652	4.9	0
2013/5/31	20.7	52	9.356231939	2.8	0
2013/6/1	20	56	9.672847543	3.1	0
2013/6/2	16.6	69	9.742161449	3.4	0
2013/6/3	13.3	76	8.775838111	5.2	0
2013/6/4	13	74	8.387831068	5	0
2013/6/5	13.2	79	9.066060844	2.6	0
2013/6/6	14.8	78	9.875478145	2.5	0
2013/6/7	18.1	64	9.883536495	2	0
2013/6/8	19.5	59	9.896735733	2.5	0
2013/6/9	17.1	73	10.62079766	3.6	0
2013/6/10	18.4	72	11.31862185	2.4	0
2013/6/11	20	67	11.57287117	3.7	0
2013/6/12	18.8	66	10.623822	5.7	0
2013/6/13	20.8	69	12.48738965	5.6	0
2013/6/14	20.6	84	15.02622758	1.4	0
2013/6/15	20	87	15.02745958	2.7	0
2013/6/16	19.8	83	14.16967864	2.1	0
2013/6/17	18.9	79	12.79168376	4.8	0
2013/6/18	18.5	81	12.80908574	3.8	0
2013/6/19	18.3	86	13.4395698	2.7	0
2013/6/20	15.9	87	11.77597805	5	0
2013/6/21	15	84	10.76558285	2.1	0
2013/6/22	14.8	89	11.26817378	2	0
2013/6/23	18.6	70	11.13527991	2.1	0
2013/6/24	19.8	73	12.46248844	2.7	0
2013/6/25	20.2	75	13.10701602	2.2	0
2013/6/26	18.8	81	13.03832701	2.6	0
2013/6/27	15.3	91	11.87751338	2.1	0
2013/6/28	15.6	76	10.10192631	3.3	0
2013/6/29	16.4	69	9.62563218	3.2	0
2013/6/30	18.9	69	11.17248328	3.6	0
2013/7/1	21.9	70	13.50088137	2.9	0
2013/7/2	22.5	70	13.97453635	4.7	1
2013/7/3	18.9	85	13.76320404	11.1	0
2013/7/4	21.1	80	14.73245967	6.4	0
2013/7/5	22.3	83	16.38074476	4	0
2013/7/6	23.2	80	16.62304028	1.6	0
2013/7/7	23.9	78	16.86552767	2.2	0
2013/7/8	24.5	74	16.55299696	2.7	0
2013/7/9	26.1	76	18.59622812	2.7	0
2013/7/10	23.1	84	17.35492145	2.8	0
2013/7/11	22.3	77	15.19659454	1.8	0
2013/7/12	21.8	76	14.57386783	1.8	0
2013/7/13	22.6	70	14.05485419	2.2	0

2013/7/14	22.4	74	14.68859209	2.5	0
2013/7/15	22.8	76	15.43525608	2.8	0
2013/7/16	22.4	79	15.68106452	2.5	0
2013/7/17	20.7	73	13.13471022	5	0
2013/7/18	20.6	63	11.26967069	4.5	0
2013/7/19	21	69	12.63326134	2.3	0
2013/7/20	22.1	71	13.85230814	2.2	0
2013/7/21	23	63	12.94210281	3.1	0
2013/7/22	21.9	73	14.07949057	4.1	0
2013/7/23	24	67	14.56939736	2.7	0
2013/7/24	22.7	75	15.1452528	6.3	0
2013/7/25	18.7	80	12.80150401	7.5	0
2013/7/26	22.2	74	14.52086161	7.5	0
2013/7/27	21.6	77	14.59621574	7.2	0
2013/7/28	22.2	81	15.89445662	4.9	0
2013/7/29	23.1	84	17.35492145	4.1	0
2013/7/30	25.3	73	17.08096077	3.9	0
2013/7/31	25.1	72	16.65893936	3	0
2013/8/1	23.1	81	16.73510283	2.2	0
2013/8/2	21.4	77	14.42848007	3.3	0
2013/8/3	20.8	75	13.57324962	3.2	0
2013/8/4	22	70	13.57884948	2.2	0
2013/8/5	23.2	71	14.75294825	5.6	0
2013/8/6	26.3	75	18.55705089	4.3	0
2013/8/7	25.5	80	18.92981365	2.4	0
2013/8/8	25.9	75	18.14798937	2.2	0
2013/8/9	23.2	88	18.28534431	6.2	0
2013/8/10	23.9	86	18.59532538	4.4	0
2013/8/11	24.2	84	18.47409386	2.4	0
2013/8/12	24.1	85	18.58853742	2	0
2013/8/13	23.4	81	17.02369411	3	0
2013/8/14	24.6	81	18.22131632	5.1	0
2013/8/15	25.7	81	19.3820914	5.8	0
2013/8/16	26.7	75	18.97400812	4.4	0
2013/8/17	25.1	87	20.12955173	5	0
2013/8/18	26	83	20.1961369	3.6	0
2013/8/19	25.9	79	19.11588214	1.9	0
2013/8/20	23.5	79	16.69810156	2.2	0
2013/8/21	22.1	81	15.80333746	2.1	0
2013/8/22	23.1	78	16.1152842	2.1	0
2013/8/23	23.7	74	15.82006611	3.2	1
2013/8/24	21.6	85	16.11270569	2.5	0
2013/8/25	21.3	84	15.64934958	2.9	0
2013/8/26	21	74	13.54871506	5	0
2013/8/27	20	81	13.99108305	3	0
2013/8/28	19.9	64	10.99018763	4.8	0
2013/8/29	20.1	65	11.29324672	2.3	0
2013/8/30	19.3	91	15.08608227	3.7	0
2013/8/31	19.1	89	14.58182828	3.7	1
2013/9/1	19.8	83	14.16967864	2.9	0

2013/9/2	20.2	85	14.85461815	4.8	0
2013/9/3	20.6	90	16.09952955	2.2	0
2013/9/4	21.3	95	17.69866917	2.3	0
2013/9/5	22.4	62	12.30665823	3.9	0
2013/9/6	18.2	79	12.27263418	2.8	0
2013/9/7	19	83	13.51887093	2	0
2013/9/8	20	78	13.47289479	2.2	0
2013/9/9	19.9	74	12.70740444	2.6	0
2013/9/10	20.5	71	12.62699593	1.7	0
2013/9/11	21.2	76	14.07718239	3.6	0
2013/9/12	21.5	77	14.51213904	1.7	0
2013/9/13	21.2	60	11.11356504	3.9	0
2013/9/14	23.4	84	17.6542013	3.5	0
2013/9/15	21.9	87	16.77966685	3.4	0
2013/9/16	17.9	88	13.42926386	2.9	0
2013/9/17	19.2	49	8.075615545	4.1	0
2013/9/18	17.6	53	7.94484499	3.5	0
2013/9/19	17.3	50	7.362082946	2.1	0
2013/9/20	19.5	64	10.73544215	4.4	0
2013/9/21	19.6	65	10.96737472	3.6	1
2013/9/22	16.7	58	8.238422674	4.2	0
2013/9/23	16.1	73	10.00110471	4.3	0
2013/9/24	18.4	76	11.94743417	3.7	0
2013/9/25	14.6	88	11.00633666	3.2	0
2013/9/26	12.8	61	6.829129374	2.9	0
2013/9/27	12.8	59	6.605223493	1.6	0
2013/9/28	15.5	63	8.323343457	4	0
2013/9/29	18.5	71	11.22771713	5.1	0
2013/9/30	17	91	13.16055757	3.3	0
2013/10/1	16.9	93	13.36941347	2.4	0
2013/10/2	19.6	79	13.3295785	3.5	0
2013/10/3	16.3	51	7.071867687	3.4	1
2013/10/4	13.5	55	6.429808257	2.4	0
2013/10/5	16.3	68	9.429156916	3.9	0
2013/10/6	16	80	10.89412817	2	0
2013/10/7	17.4	79	11.70180475	5.5	0
2013/10/8	17.1	65	9.456874626	3.6	0
2013/10/9	12.8	78	8.732329363	1.7	0
2013/10/10	14.8	72	9.11582598	1.9	0
2013/10/11	17	90	13.01593606	5.1	0
2013/10/12	16.2	59	8.132004503	5	0
2013/10/13	12.1	61	6.538176232	4.7	0
2013/10/14	14	60	7.233506441	3.6	0
2013/10/15	12.3	66	7.162825632	4.7	1
2013/10/16	6.9	72	5.539780102	4.7	0
2013/10/17	8.2	65	5.44069959	2.4	0
2013/10/18	9.7	61	5.620658634	2.4	0
2013/10/19	9.6	70	6.409004994	1.7	0
2013/10/20	11.4	77	7.899488587	3	0
2013/10/21	12.6	74	8.182309179	1.8	0

2013/10/22	11.7	64	6.690406927	1.8	0
2013/10/23	11.4	76	7.796897826	2.6	0
2013/10/24	11.6	76	7.895269724	3.9	1
2013/10/25	13	90	10.20141616	6.3	0
2013/10/26	11	69	6.903032828	4.6	0
2013/10/27	8.6	74	6.355484859	6.4	0
2013/10/28	8.5	79	6.741455644	2.2	0
2013/10/29	11.1	72	7.248632723	2.7	0
2013/10/30	12	78	8.308281481	2.3	0
2013/10/31	11.2	61	6.179935127	2.5	0
2013/11/1	10.1	48	4.536644834	4.4	0
2013/11/2	9.9	60	5.599268757	1.9	0
2013/11/3	11.4	73	7.489125543	2.2	0
2013/11/4	9.1	71	6.2963981	2.4	0
2013/11/5	9.9	68	6.345837925	1.5	0
2013/11/6	12.1	65	6.9669091	4.1	0
2013/11/7	12.6	76	8.403452671	3.4	0
2013/11/8	5	82	5.569045303	6.2	0
2013/11/9	6.5	57	4.272658274	4.1	0
2013/11/10	6.7	64	4.860449446	6.4	0
2013/11/11	0.3	65	3.213544148	2.6	0
2013/11/12	0.8	62	3.172480968	2.3	0
2013/11/13	1.6	69	3.729243627	2.4	0
2013/11/14	5	59	4.006996011	2.5	0
2013/11/15	8.3	75	6.318297444	4	0
2013/11/16	6.5	69	5.172165279	1.3	0
2013/11/17	5.4	71	4.951160591	2.8	0
2013/11/18	8.6	80	6.870794442	3.6	0
2013/11/19	7.3	83	6.554455588	1.6	0
2013/11/20	5.7	62	4.409873293	2.5	0
2013/11/21	7	66	5.111309356	2.9	0
2013/11/22	6.1	70	5.111474577	2.8	0
2013/11/23	6.5	70	5.247124196	1.9	0
2013/11/24	5.4	65	4.532752654	2.1	0
2013/11/25	7.7	75	6.078273888	7.6	0
2013/11/26	7.9	52	4.26912014	4.2	0
2013/11/27	2.5	65	3.734432922	2.6	0
2013/11/28	0.4	79	3.932696826	2.7	0
2013/11/29	0.5	64	3.207991585	2.4	0
2013/11/30	2.2	69	3.884513927	2.5	0
2013/12/1	1.6	62	3.350914563	2.7	0
2013/12/2	2.3	80	4.53441585	2.4	0
2013/12/3	1.9	76	4.19232233	1.6	0
2013/12/4	4.3	67	4.343644938	2.6	0
2013/12/5	6.5	63	4.722411777	2.5	0
2013/12/6	2.9	75	4.42692535	2.1	0
2013/12/7	1.1	61	3.186163427	3.4	0
2013/12/8	1.5	64	3.435500911	2.9	0
2013/12/9	3.1	59	3.529739703	2.9	0
2013/12/10	7.1	90	7.015466524	3.4	0

2013/12/11	3.5	64	3.933146038	5.4	0
2013/12/12	-0.1	66	3.174088347	2.5	0
2013/12/13	-2.7	81	3.247581377	3.2	0
2013/12/14	-3.3	52	1.997978613	4.3	0
2013/12/15	-0.6	78	3.62339895	7.7	0
2013/12/16	2.9	75	4.42692535	5.2	0
2013/12/17	2	66	3.665526706	3.1	0
2013/12/18	1.2	70	3.681358136	1.8	0
2013/12/19	-0.2	67	3.199958929	1.9	0
2013/12/20	0.2	61	2.995061131	1.9	0
2013/12/21	-0.1	55	2.645073623	3.6	0
2013/12/22	-2.9	77	3.043785648	2.5	0
2013/12/23	-1.6	83	3.595797726	2.9	0
2013/12/24	-2.7	69	2.76645821	1.7	0
2013/12/25	-0.9	74	3.366585717	2	0
2013/12/26	2.3	67	3.797573274	2.7	0
2013/12/27	2.1	76	4.249666829	4.3	0
2013/12/28	-1.8	76	3.246669419	5.7	0
2013/12/29	-3	72	2.826024609	3.4	0
2013/12/30	-2	88	3.706839667	1.5	0
2013/12/31	-0.6	71	3.298222121	2.3	0
2014/1/1	-0.3	82	3.889335145	3.6	0
2014/1/2	-3.4	66	2.517912755	5.2	0
2014/1/3	-1.7	70	3.0114121	3.4	0
2014/1/4	-3.7	73	2.726032371	3.2	0
2014/1/5	-4.2	73	2.630239654	2.7	0
2014/1/6	-3.7	67	2.501974916	3.3	0
2014/1/7	0.1	61	2.974459322	2.9	0
2014/1/8	0.4	73	3.634010991	3.3	0
2014/1/9	-5.1	56	1.891135634	4.1	0
2014/1/10	-7	47	1.381933059	4.5	0
2014/1/11	-6.6	59	1.786438625	3.3	0
2014/1/12	-6.2	74	2.30711553	3.5	0
2014/1/13	-7.6	65	1.828506191	3.8	0
2014/1/14	-4.6	55	1.925560267	2.8	0
2014/1/15	-4.7	64	2.224579798	1.9	0
2014/1/16	-5.2	62	2.07867241	2.2	0
2014/1/17	-6.5	78	2.379086991	1.8	0
2014/1/18	-6	68	2.151197823	1.7	0
2014/1/19	-5.7	75	2.425003187	1.9	0
2014/1/20	-7	74	2.175809497	1.3	0
2014/1/21	-6.3	80	2.476033236	2	0
2014/1/22	-5.4	75	2.478373093	3	0
2014/1/23	-8.1	75	2.033107037	2.2	0
2014/1/24	-1.8	82	3.502985426	4.2	0
2014/1/25	1.7	70	3.80915569	3	0
2014/1/26	-2.6	85	3.432145913	4.6	0
2014/1/27	-4.9	62	2.1242155	4.2	0
2014/1/28	-2.1	77	3.220743749	3.7	0
2014/1/29	-6.1	54	1.695897888	5.5	0

2014/1/30	1.6	73	3.945431663	4.8	0
2014/1/31	-3.1	71	2.767063892	5.8	0
2014/2/1	-5.1	59	1.992446472	4.1	0
2014/2/2	-2.2	71	2.94893661	4.6	0
2014/2/3	-1.5	59	2.574017666	3.1	0
2014/2/4	-6.4	70	2.150753095	2.6	0
2014/2/5	-8.5	69	1.815635705	2.4	0
2014/2/6	-7.7	53	1.479952901	3.3	0
2014/2/7	-7.6	63	1.772244462	3.9	1
2014/2/8	-9.2	61	1.523310408	1.7	0
2014/2/9	-6.6	68	2.058946212	1.6	0
2014/2/10	-3.2	79	3.057050505	1.9	0
2014/2/11	-4	80	2.92405104	1.7	0
2014/2/12	-4.4	69	2.450688142	2.1	0
2014/2/13	-3.7	73	2.726032371	3.1	0
2014/2/14	-4.7	56	1.946507323	2	0
2014/2/15	-4.1	68	2.467709299	2.1	0
2014/2/16	-1	85	3.840169707	5.7	0
2014/2/17	-1.9	77	3.266366132	9.3	0
2014/2/18	-2.9	77	3.043785648	8.6	0
2014/2/19	-1.9	79	3.351206811	6.8	0
2014/2/20	-3	64	2.512021875	2.6	0
2014/2/21	-3.7	82	3.062118554	3.2	0
2014/2/22	-2.8	76	3.025618202	2.2	0
2014/2/23	-2.8	76	3.025618202	1.8	0
2014/2/24	-2.6	66	2.664960356	1.4	0
2014/2/25	-0.6	67	3.112406791	2	0
2014/2/26	1.2	64	3.365813153	2.3	0
2014/2/27	2.1	61	3.410916797	3.9	0
2014/2/28	0.7	58	2.947495947	6	1
2014/3/1	-0.8	42	1.924115228	3.3	0
2014/3/2	-2.6	47	1.897774799	3.9	0
2014/3/3	-4	63	2.302690194	2.6	0
2014/3/4	-3	63	2.472771533	3.7	0
2014/3/5	-1.7	88	3.785775211	3.2	0
2014/3/6	-4.2	73	2.630239654	7.4	0
2014/3/7	-5.7	65	2.101669428	2.4	0
2014/3/8	-4.5	65	2.29208875	2	0
2014/3/9	-2.3	71	2.92822469	2	0
2014/3/10	-1.9	71	3.011844096	5	0
2014/3/11	-2.7	75	3.007019793	2	0
2014/3/12	-2.1	73	3.053432385	1.8	0
2014/3/13	-0.4	70	3.297227052	2.1	0
2014/3/14	-0.1	65	3.125996099	6	0
2014/3/15	-1	71	3.207671167	2.7	0
2014/3/16	0.6	80	4.037667151	3.1	0
2014/3/17	1.7	63	3.428240121	3.3	0
2014/3/18	2.1	77	4.305583498	6.2	0
2014/3/19	1.5	63	3.38182121	4.3	0
2014/3/20	0.9	62	3.19432102	3.1	0

2014/3/21	-0.8	76	3.481732316	5.3	0
2014/3/22	0	79	3.825648205	3.7	0
2014/3/23	0.9	62	3.19432102	2.7	0
2014/3/24	4.8	57	3.820200937	1.9	0
2014/3/25	7.6	63	5.072802914	3	0
2014/3/26	5.8	74	5.298137279	2.1	0
2014/3/27	5.2	69	4.748558056	1.6	0
2014/3/28	5.3	73	5.05713726	2.9	0
2014/3/29	6.8	64	4.892258685	3.2	0
2014/3/30	4.2	71	4.572398839	3.4	0
2014/3/31	4.4	66	4.307395407	6.2	0
2014/4/1	4.7	57	3.794938623	5.9	0
2014/4/2	6.4	54	4.021396426	2.8	0
2014/4/3	7.2	67	5.256688987	4.5	0
2014/4/4	2.4	87	4.964686723	5.9	0
2014/4/5	1.6	66	3.567102599	5.8	0
2014/4/6	2.7	58	3.377607264	3.3	0
2014/4/7	4.5	58	3.810549646	4.3	0
2014/4/8	3.9	51	3.219287943	2.9	0
2014/4/9	8.5	41	3.498730144	3.2	0
2014/4/10	3.9	72	4.544877096	5.8	0
2014/4/11	2.9	50	2.951283567	6.3	0
2014/4/12	4.5	49	3.21925746	6.1	0
2014/4/13	4.1	60	3.838316465	4.6	1
2014/4/14	7.7	39	3.160702422	1.9	0
2014/4/15	9.1	42	3.724629862	3.7	0
2014/4/16	6	33	2.393934678	5.3	0
2014/4/17	5	43	2.920353025	4	0
2014/4/18	4.5	56	3.679151382	5.1	0
2014/4/19	3.6	52	3.217174072	3.5	0
2014/4/20	6	56	4.062434604	2.1	0
2014/4/21	8.6	48	4.122476665	3	1
2014/4/22	8.5	65	5.546767302	2.9	0
2014/4/23	8.1	73	6.071059454	3.9	0
2014/4/24	12.5	53	5.823995438	1.9	0
2014/4/25	14.5	42	5.221009049	1.8	0
2014/4/26	16.4	36	5.022068963	1.9	0
2014/4/27	17.9	31	4.730763405	3.6	0
2014/4/28	10.3	57	5.455979031	5.1	0
2014/4/29	9.6	40	3.662288568	3	0
2014/4/30	12.8	52	5.821552909	4	0
2014/5/1	14.2	76	9.27557369	3.1	0
2014/5/2	12.9	79	8.899281231	2.8	0
2014/5/3	13.2	68	7.803697942	5.3	0
2014/5/4	11.7	42	4.390579546	3.2	0
2014/5/5	12.4	55	6.00629427	5.6	0
2014/5/6	11.9	48	5.08095565	3.7	0
2014/5/7	12.5	50	5.494335319	4.1	0
2014/5/8	14.5	45	5.593938267	3.6	0
2014/5/9	12.6	77	8.514024416	4	0

2014/5/10	11.8	73	7.679135802	4.7	0
2014/5/11	15.5	50	6.605828141	2.1	0
2014/5/12	18.2	39	6.058642188	4.7	0
2014/5/13	15	65	8.33051054	4.4	0
2014/5/14	16.7	64	9.090673296	3.2	0
2014/5/15	14.6	74	9.255328551	2.2	0
2014/5/16	10.3	91	8.710422663	4	0
2014/5/17	7	81	6.272970573	9.5	0
2014/5/18	8.5	79	6.741455644	6	0
2014/5/19	12.3	67	7.271353293	3	0
2014/5/20	14.9	61	7.770372055	2.2	0
2014/5/21	15.7	77	10.2970428	3.8	0
2014/5/22	14.1	65	7.88454898	2.7	0
2014/5/23	11.3	63	6.422777051	3	0
2014/5/24	12.7	68	7.565712969	3	0
2014/5/25	11.4	83	8.515033152	3.6	0
2014/5/26	12.1	73	7.824374835	9.5	0
2014/5/27	16.2	71	9.785971521	6	0
2014/5/28	18.9	63	10.20096299	4.5	0
2014/5/29	20.8	54	9.77273973	5.2	0
2014/5/30	18.9	65	10.52480309	3.8	0
2014/5/31	20	60	10.36376522	2.1	0
2014/6/1	17.9	54	8.240684641	3.8	0
2014/6/2	21.5	44	8.292650879	2.2	0
2014/6/3	23.9	45	9.730112116	1.9	0
2014/6/4	23.9	47	10.16256154	2.5	0
2014/6/5	21.8	47	9.012786685	7.8	0
2014/6/6	18.8	62	9.979954004	7.4	0
2014/6/7	18	67	10.28553324	6.8	0
2014/6/8	18.1	82	12.66328113	4.6	0
2014/6/9	19.7	80	13.57771308	5.6	0
2014/6/10	19.6	76	12.82339198	4.7	0
2014/6/11	18.7	78	12.48146641	5.9	0
2014/6/12	17.9	83	12.6662375	5.3	0
2014/6/13	15.7	92	12.30296022	2.7	0
2014/6/14	15	79	10.12477435	2.3	0
2014/6/15	16.2	88	12.12909146	1.6	0
2014/6/16	15.7	92	12.30296022	1.5	0
2014/6/17	17	85	12.2928285	1.8	0
2014/6/18	16.9	87	12.50687067	1.8	0
2014/6/19	15.6	86	11.43112714	3.4	0
2014/6/20	14.9	82	10.44541817	4.8	0
2014/6/21	15.3	80	10.44177	3.7	0
2014/6/22	16.6	79	11.15406891	3.2	0
2014/6/23	16.6	76	10.73049667	4.6	0
2014/6/24	17.3	73	10.7486411	2.8	0
2014/6/25	20	70	12.09105943	2.2	0
2014/6/26	21.3	67	12.48221931	3	0
2014/6/27	22.6	68	13.65328693	6.3	0
2014/6/28	21.9	73	14.07949057	6.4	0

2014/6/29	21.8	69	13.2315379	5.9	0
2014/6/30	21.2	65	12.03969546	2.4	0
2014/7/1	21.2	70	12.96582588	2.7	0
2014/7/2	21.4	75	14.05371435	2.1	0
2014/7/3	21.6	75	14.21709326	2.5	0
2014/7/4	22.3	66	13.02565246	3.6	0
2014/7/5	20.2	65	11.35941388	2.2	0
2014/7/6	19.8	72	12.29176942	1.6	0
2014/7/7	21.8	72	13.80682216	1.8	0
2014/7/8	21.9	74	14.27236031	2.6	0
2014/7/9	21.3	73	13.60002999	5.2	0
2014/7/10	20.3	83	14.59001478	4.9	0
2014/7/11	21	80	14.64725952	4.3	0
2014/7/12	19.7	77	13.06854884	3.2	0
2014/7/13	22.5	75	14.97271752	2.2	0
2014/7/14	24	69	15.00430474	3.3	0
2014/7/15	25.5	57	13.48749222	2.6	0
2014/7/16	24.3	63	13.93413561	2	0
2014/7/17	24.2	71	15.6150079	2.4	0
2014/7/18	23.9	65	14.05460639	4.8	0
2014/7/19	21.9	67	12.92227217	6.6	0
2014/7/20	21.9	65	12.5365327	6.2	0
2014/7/21	22.9	58	11.84707807	6.6	0
2014/7/22	22.6	73	14.65720508	5.6	0
2014/7/23	22.9	85	17.36209717	2	0
2014/7/24	25.7	62	14.8356749	2.8	0
2014/7/25	25	50	11.50388899	2.6	1
2014/7/26	21.4	76	14.24109721	3.1	0
2014/7/27	21.5	86	16.20836308	5.4	0
2014/7/28	20.6	76	13.59515829	5	1
2014/7/29	23.9	61	13.18970754	2	0
2014/7/30	24.9	59	13.49847147	2.1	0
2014/7/31	26.1	63	15.41529436	2.4	0
2014/8/1	25.4	70	16.47107295	2.6	1
2014/8/2	25.1	69	15.96481689	2.3	0
2014/8/3	25.8	72	17.32506549	2.7	0
2014/8/4	27	74	19.03475617	3.5	0
2014/8/5	24.9	90	20.59088869	2.5	1
2014/8/6	23.3	88	18.38985404	1.9	0
2014/8/7	22.4	87	17.26902043	1.9	0
2014/8/8	22.6	82	16.46425777	1.9	0
2014/8/9	23.3	73	15.25521983	1.9	0
2014/8/10	21.7	74	14.10873612	4.4	0
2014/8/11	23.5	79	16.69810156	6.8	0
2014/8/12	23.9	59	12.75725811	4.8	0
2014/8/13	23.3	62	12.95648808	2.9	0
2014/8/14	21.7	67	12.77412594	2.6	0
2014/8/15	20.9	74	13.47029819	1.7	0
2014/8/16	22	71	13.77283304	2.1	0
2014/8/17	22.3	67	13.22301083	3.4	0

2014/8/18	22.1	76	14.8278228	3.6	0
2014/8/19	20.6	88	15.74176223	4.3	0
2014/8/20	22	85	16.48860293	4	0
2014/8/21	22.7	73	14.74137939	2.3	0
2014/8/22	21.1	83	15.2849269	5	0
2014/8/23	23.8	78	16.77013391	4.6	0
2014/8/24	24	66	14.35194367	2.8	0
2014/8/25	19.3	74	12.26780316	3	0
2014/8/26	17.9	57	8.698500454	3.1	0
2014/8/27	18.6	66	10.4989782	2.1	0
2014/8/28	20	69	11.91833001	1.8	0
2014/8/29	20.6	61	10.91190336	2.7	0
2014/8/30	21.1	57	10.49687751	3.7	0
2014/8/31	20.9	63	11.46795657	2.1	0
2014/9/1	21.1	61	11.2335005	3	0
2014/9/2	21.8	64	12.27273081	3.2	1
2014/9/3	21.6	67	12.70060331	3.7	0
2014/9/4	18.7	84	13.44157921	6.9	0
2014/9/5	22.3	80	15.78866965	2.9	0
2014/9/6	23	54	11.09323098	2.8	0
2014/9/7	20.2	71	12.40797516	2.5	0
2014/9/8	20.3	71	12.48061506	2.8	0
2014/9/9	21	70	12.81635208	3.5	0
2014/9/10	19.8	77	13.14536452	4.9	0
2014/9/11	18.6	80	12.72603418	4.4	0
2014/9/12	18.6	75	11.93065704	2.2	0
2014/9/13	18.4	65	10.21820028	3.3	0
2014/9/14	15.7	63	8.424853197	2.4	0
2014/9/15	17.3	65	9.570707829	1.4	0
2014/9/16	17.4	66	9.776191309	3.8	0
2014/9/17	15	60	7.689702037	2.8	0
2014/9/18	14.8	66	8.356173815	3.5	0
2014/9/19	15.4	74	9.71747647	4.4	0
2014/9/20	15.4	73	9.58615922	2.1	0
2014/9/21	16.4	59	8.230613023	1.8	0
2014/9/22	16.5	66	9.262712829	2	0
2014/9/23	17.6	63	9.443872347	1.5	0
2014/9/24	18.1	66	10.19239701	2.7	0
2014/9/25	18.3	72	11.25173286	4.6	0
2014/9/26	16	57	7.762066324	3.7	0
2014/9/27	15.5	64	8.45546002	5.6	0
2014/9/28	18.7	64	10.24120321	5.5	0
2014/9/29	15.2	59	7.654139076	1.8	0
2014/9/30	13	71	8.047783862	3.6	0
2014/10/1	12.2	63	6.794778191	2	0
2014/10/2	13.7	62	7.338013895	2.8	0
2014/10/3	13.4	87	10.10823959	2.8	0
2014/10/4	13	73	8.274481999	3.5	0
2014/10/5	13.2	54	6.197054248	2.8	0
2014/10/6	10.8	69	6.816597014	1.8	0

2014/10/7	11	60	6.002637242	2.4	0
2014/10/8	12.7	66	7.343192	2	1
2014/10/9	14.3	61	7.490633126	2.6	0
2014/10/10	12.6	61	6.744876486	6.6	0
2014/10/11	12.2	55	5.931949215	6.7	0
2014/10/12	12.1	66	7.074092317	2.7	0
2014/10/13	12.2	62	6.686924569	3	0
2014/10/14	8.5	78	6.656120763	2.5	0
2014/10/15	9.9	61	5.692589903	2.4	0
2014/10/16	10.9	73	7.257360319	4.5	0
2014/10/17	11.4	78	8.002079347	3.1	0
2014/10/18	10.9	51	5.070210634	4.2	0
2014/10/19	11.9	57	6.033634835	2.6	0
2014/10/20	15.6	62	8.241045145	3.7	0
2014/10/21	10.5	70	6.785642997	3.8	0
2014/10/22	6.2	53	3.895573753	2.6	0
2014/10/23	6.8	56	4.28072635	1.9	0
2014/10/24	10.1	59	5.576292608	2.1	0
2014/10/25	14.2	69	8.421244535	3.9	0
2014/10/26	17.7	63	9.500333725	5.5	0
2014/10/27	9.4	73	6.599011514	4	0
2014/10/28	4.9	68	4.58774491	3.7	0
2014/10/29	7.3	52	4.106405911	5.1	0
2014/10/30	8.6	55	4.723671179	1.5	0
2014/10/31	10.7	73	7.16641272	1.5	0
2014/11/1	11.3	72	7.34031663	3	0
2014/11/2	14.2	87	10.61809093	3.9	0
2014/11/3	6.5	65	4.872329611	5	0
2014/11/4	6.4	54	4.021396426	4.2	0
2014/11/5	10.9	55	5.467874213	3.4	0
2014/11/6	11.2	70	7.091728834	4	0
2014/11/7	4.2	49	3.155599199	5	0
2014/11/8	8.7	47	4.062588791	2.6	0
2014/11/9	10.2	62	5.897100461	1.8	0
2014/11/10	9.1	80	7.09453307	3.7	0
2014/11/11	7.8	67	5.465161517	2.6	0
2014/11/12	9.3	75	6.736674198	5.3	0
2014/11/13	4.6	61	4.034370967	4.3	1
2014/11/14	-0.4	86	4.050878949	2.4	0
2014/11/15	0.1	72	3.510837233	3.2	0
2014/11/16	2.8	59	3.459109538	2.7	0
2014/11/17	2.3	60	3.400811887	1.6	0
2014/11/18	1.9	83	4.578457281	1.5	0
2014/11/19	3.8	57	3.574051805	2.9	0
2014/11/20	3.7	62	3.861637779	1.8	0
2014/11/21	6.4	65	4.840569772	3.6	0
2014/11/22	8.7	75	6.482854454	2.9	0
2014/11/23	3.8	53	3.323241152	4.4	0
2014/11/24	4.4	67	4.372658973	2.5	1
2014/11/25	3.7	52	3.238792976	4.7	0

2014/11/26	2.9	61	3.600565951	3.3	0
2014/11/27	5.5	72	5.054119541	2.5	0
2014/11/28	6.4	67	4.98951038	1.6	0
2014/11/29	6.4	71	5.287391597	2.3	0
2014/11/30	5.3	76	5.264964819	2.4	0
2014/12/1	8.8	75	6.524571194	6	0
2014/12/2	0.1	47	2.291796527	5.1	0
2014/12/3	-1.3	52	2.300591928	3.8	0
2014/12/4	-1.4	61	2.6799645	1.9	0
2014/12/5	-2.4	56	2.293350075	1.9	0
2014/12/6	-3.5	52	1.969729615	2.6	0
2014/12/7	-1.9	63	2.672481381	3.9	0
2014/12/8	-1.6	68	2.945954764	2.1	0
2014/12/9	-2	73	3.074991997	2.5	0
2014/12/10	-0.9	63	2.866147299	2.7	0
2014/12/11	1.6	79	4.269713718	4.5	0
2014/12/12	-1.8	64	2.734037405	2.9	0
2014/12/13	-4.8	74	2.553703991	1.9	0
2014/12/14	-4.8	53	1.829004209	2.4	0
2014/12/15	-3.7	88	3.286176009	1.3	0
2014/12/16	-1.2	83	3.697845254	5.5	0
2014/12/17	-0.6	60	2.787229962	3.7	0
2014/12/18	1	64	3.320043712	5.4	0
2014/12/19	-0.2	75	3.582043577	1.9	0
2014/12/20	3.7	69	4.297629141	6.6	1
2014/12/21	-2.5	53	2.155220885	3.3	0
2014/12/22	-6.5	70	2.135078069	2.3	0
2014/12/23	-3.3	58	2.228514607	3.1	0
2014/12/24	-2.4	88	3.603835832	2.3	0
2014/12/25	-5	74	2.517119747	2.3	0
2014/12/26	-4.4	70	2.486205361	3.1	0
2014/12/27	-2	66	2.78012975	2.7	0
2014/12/28	1.2	69	3.628767305	2.4	0
2014/12/29	1.7	76	4.135654749	2.4	0
2014/12/30	0.2	75	3.68245221	2	0
2014/12/31	-1.2	70	3.118664672	2	0
2015/1/1	-4	64	2.339240832	1.9	0
2015/1/2	-5.5	65	2.1324071	2.3	0
2015/1/3	-5	52	1.768786849	3	0
2015/1/4	-2.7	67	2.686271015	1.8	0
2015/1/5	-2.4	72	2.948592954	1.9	0
2015/1/6	1.1	75	3.917414049	5.1	0
2015/1/7	-3.4	77	2.93756488	7.9	0
2015/1/8	0.7	81	4.116330546	9.5	0
2015/1/9	0.7	77	3.913054964	6.2	0
2015/1/10	-0.9	65	2.957136102	4.1	0
2015/1/11	0.2	70	3.436955396	5.2	0
2015/1/12	1	76	3.942551908	5.3	0
2015/1/13	-0.1	65	3.125996099	4.2	0
2015/1/14	-1.1	65	2.916189514	1.7	0

2015/1/15	-0.4	70	3.297227052	1.4	0
2015/1/16	-1.3	70	3.096950672	1.7	0
2015/1/17	-3.9	80	2.945046002	5.6	0
2015/1/18	-4.9	70	2.398307822	5.4	0
2015/1/19	-3	70	2.747523926	2.1	0
2015/1/20	-3.8	74	2.743710025	4	0
2015/1/21	-2.2	67	2.782799337	1.8	0
2015/1/22	0.1	77	3.754645374	2.7	0
2015/1/23	0.2	87	4.271644563	5.7	0
2015/1/24	-0.6	74	3.43758362	3.2	0
2015/1/25	-1.1	73	3.275105147	1.7	0
2015/1/26	1.9	77	4.247484466	4.6	0
2015/1/27	0.9	71	3.658012781	6.3	0
2015/1/28	-4.9	51	1.747338556	3	0
2015/1/29	-2.9	53	2.095073238	2.2	0
2015/1/30	-0.4	70	3.297227052	3.6	0
2015/1/31	-0.3	65	3.083009567	3.2	0
2015/2/1	-1.7	73	3.140472618	7.7	0
2015/2/2	-2.1	74	3.095260226	8.7	0
2015/2/3	-4.4	59	2.095515947	5	0
2015/2/4	-5.9	57	1.816389466	2.3	0
2015/2/5	-5.2	63	2.112199384	1.7	0
2015/2/6	-4.1	63	2.286260085	2.7	0
2015/2/7	0.2	67	3.289657307	2.7	0
2015/2/8	2.4	83	4.736425265	8.2	0
2015/2/9	-4.9	48	1.644553935	4.5	0
2015/2/10	-6.7	60	1.803453172	2.8	0
2015/2/11	-2.7	68	2.726364612	2.9	0
2015/2/12	-1.5	76	3.315683774	2	0
2015/2/13	-2.7	87	3.48814296	2.7	0
2015/2/14	-0.9	70	3.18460811	6.5	0
2015/2/15	0.6	69	3.482487917	10.7	0
2015/2/16	1.8	76	4.163904348	7.3	0
2015/2/17	1.9	69	3.806187378	2.4	0
2015/2/18	1.7	62	3.373823611	3.5	0
2015/2/19	0.8	67	3.428326208	2.4	0
2015/2/20	0.3	55	2.71915274	2.8	0
2015/2/21	-0.1	58	2.789350366	2.2	0
2015/2/22	1.8	77	4.218692563	7.5	0
2015/2/23	4.3	61	3.95466181	4.2	0
2015/2/24	0.7	70	3.557322694	2.8	0
2015/2/25	-0.3	74	3.509887814	1.5	0
2015/2/26	0.6	68	3.432017078	3.7	0
2015/2/27	2.3	79	4.477735652	5.7	0
2015/2/28	0.3	73	3.609057273	5.4	0
2015/3/1	-0.3	78	3.69961148	4.4	0
2015/3/2	1.5	76	4.079657332	6.1	0
2015/3/3	2.3	53	3.004050501	3.1	0
2015/3/4	1	85	4.409433055	2.5	0
2015/3/5	0.8	81	4.144692878	3.6	0

2015/3/6	1.4	74	3.945278685	2.2	0
2015/3/7	1.5	85	4.562774648	5.1	0
2015/3/8	3.2	66	3.975170045	1.8	0
2015/3/9	4.7	74	4.926762423	7.2	0
2015/3/10	3.6	87	5.382579697	4.8	0
2015/3/11	0.5	65	3.258116454	4.1	0
2015/3/12	0.8	61	3.12131192	2.8	0
2015/3/13	0.9	83	4.276268462	3.1	0
2015/3/14	2.2	71	3.997108533	2.1	0
2015/3/15	2.9	57	3.364463266	2.6	0
2015/3/16	6	60	4.352608505	3.7	0
2015/3/17	6.7	69	5.240172059	3.5	0
2015/3/18	5.3	57	3.948723614	3.2	0
2015/3/19	4.7	59	3.928094364	2	0
2015/3/20	3.9	73	4.608000389	2.5	0
2015/3/21	5	57	3.871165638	2	0
2015/3/22	5.4	64	4.463017998	2.7	0
2015/3/23	2.1	53	2.963583447	4	0
2015/3/24	1.6	61	3.296867554	5.5	0
2015/3/25	2.8	64	3.752254414	7.9	0
2015/3/26	5.4	47	3.277528842	2.2	0
2015/3/27	8.3	58	4.886150023	1.8	0
2015/3/28	10	54	5.071444915	2.4	0
2015/3/29	8.6	66	5.668405414	3.7	0
2015/3/30	8.5	63	5.376097539	2.3	0
2015/3/31	7.7	66	5.348881022	3.5	0
2015/4/1	5.4	66	4.60248731	3.2	0
2015/4/2	6.9	47	3.616245344	1.9	0
2015/4/3	6.4	77	5.734213422	8.4	0
2015/4/4	7	53	4.104536301	4.8	0
2015/4/5	6.7	67	5.088283014	9.4	0
2015/4/6	8.2	69	5.775511872	4	0
2015/4/7	2	48	2.665837604	5.3	0
2015/4/8	2.2	42	2.364486738	3.9	0
2015/4/9	4	43	2.732494408	3	0
2015/4/10	5.5	66	4.632942913	6	0
2015/4/11	8.4	69	5.850364972	4	0
2015/4/12	7.9	54	4.433317068	3.2	0
2015/4/13	6.8	51	3.89851864	4	0
2015/4/14	10.8	66	6.520223231	4.9	0
2015/4/15	8.7	84	7.260796988	2	0
2015/4/16	5.9	76	5.477214173	2.7	0
2015/4/17	6	73	5.295673681	8	0
2015/4/18	9	59	5.198848131	3.6	0
2015/4/19	7.3	65	5.133007388	5	0
2015/4/20	6.6	76	5.734224227	5.6	0
2015/4/21	7.9	75	6.157384817	4.7	0
2015/4/22	11.1	56	5.637825452	2.3	1
2015/4/23	12.9	41	4.61861431	3.4	0
2015/4/24	11.4	55	5.642491848	5.1	0

2015/4/25	11.6	36	3.739864606	2.9	0
2015/4/26	15.7	27	3.61065137	4.1	0
2015/4/27	15.2	53	6.875752051	4.3	0
2015/4/28	12.7	71	7.899494424	2.6	1
2015/4/29	14.7	53	6.669430949	2.3	0
2015/4/30	16.1	49	6.713070284	2.4	0
2015/5/1	13.7	51	6.036108204	2.9	0
2015/5/2	16	30	4.085298066	5.5	0
2015/5/3	17.1	48	6.983538185	3.1	0
2015/5/4	15	70	8.971319043	5.8	0
2015/5/5	15.3	35	4.568274375	4.9	0
2015/5/6	15.8	35	4.708893626	5.1	0
2015/5/7	14	30	3.616753221	3	0
2015/5/8	12.4	42	4.586624715	3.3	0
2015/5/9	9.8	65	6.027444609	4.1	0
2015/5/10	10.3	61	5.838854752	4	0
2015/5/11	15.3	34	4.43775225	3.1	0
2015/5/12	13.6	49	5.763791562	4.5	0
2015/5/13	13.7	75	8.876629712	2.9	0
2015/5/14	9.7	80	7.371355586	4.5	0
2015/5/15	9.6	69	6.31744778	7.2	0
2015/5/16	10.4	80	7.706133581	3.5	0
2015/5/17	11.6	55	5.713682037	3.8	0
2015/5/18	14.9	43	5.477475383	3.3	0
2015/5/19	13.4	75	8.71399965	8.7	0
2015/5/20	12.5	70	7.692069446	2.2	0
2015/5/21	11.4	77	7.899488587	4.1	0
2015/5/22	15.2	62	8.043332588	3.9	0
2015/5/23	17.8	46	6.978190182	4.1	0
2015/5/24	14.5	48	5.966867485	4.1	0
2015/5/25	14.7	37	4.656017832	7.7	0
2015/5/26	13.9	73	8.746864834	4.5	0
2015/5/27	14.4	61	7.536641346	3.9	0
2015/5/28	15.7	61	8.15739754	6.6	1
2015/5/29	20.2	56	9.786571958	4.1	0
2015/5/30	20.6	68	12.164089	5.5	0
2015/5/31	17.9	61	9.308921539	3.7	0
2015/6/1	20.5	36	6.402420473	3.6	0
2015/6/2	18.4	58	9.117778709	3.7	0
2015/6/3	17.6	73	10.9428997	3.8	0
2015/6/4	12.4	76	8.299606627	5.7	0
2015/6/5	10.4	80	7.706133581	6.5	1
2015/6/6	11.5	65	6.710351971	5.9	0
2015/6/7	13	63	7.140991314	4	1
2015/6/8	17	62	8.966533729	3.8	0
2015/6/9	15.5	80	10.56932502	8.3	0
2015/6/10	15.4	79	10.37406272	3.9	0
2015/6/11	13.9	85	10.18470563	4	0
2015/6/12	17.4	66	9.776191309	4.3	1
2015/6/13	17.6	77	11.54251065	4.4	0

2015/6/14	17.9	75	11.44539533	1.9	0
2015/6/15	17.7	79	11.91311689	2.3	0
2015/6/16	18	79	12.1277183	2.2	0
2015/6/17	21	65	11.90089836	4.8	0
2015/6/18	20.2	57	9.961332172	5.2	0
2015/6/19	19	65	10.5870676	4.7	0
2015/6/20	16.6	77	10.87168741	4.3	0
2015/6/21	18.4	66	10.37540336	2.8	0
2015/6/22	17.4	77	11.40555653	2.2	0
2015/6/23	16.7	88	12.49967578	2.4	0
2015/6/24	16.4	83	11.578659	1.9	1
2015/6/25	17.4	80	11.84992886	1.8	0
2015/6/26	19.6	74	12.48593429	2.7	0
2015/6/27	17.8	70	10.61898506	2.9	0
2015/6/28	15.1	77	9.92871639	1.5	1
2015/6/29	15.5	84	11.09779128	1.8	0
2015/6/30	15.8	86	11.57042434	4.7	0
2015/7/1	17	84	12.14820699	4.8	0
2015/7/2	18.6	76	12.08973247	4.3	0
2015/7/3	17.8	69	10.46728527	3.9	0
2015/7/4	16.2	76	10.47512445	2.6	0
2015/7/5	18.1	71	10.9645483	3.2	0
2015/7/6	19.1	71	11.63269447	2.5	0
2015/7/7	16.7	74	10.511091	5.2	0
2015/7/8	15.7	63	8.424853197	4.3	0
2015/7/9	18.2	62	9.631687581	2.1	0
2015/7/10	19.6	66	11.13610356	2.2	0
2015/7/11	22	62	12.02698096	2	0
2015/7/12	24.8	60	13.65022499	1.8	0
2015/7/13	23.8	61	13.11510472	3.6	0
2015/7/14	25.5	68	16.0903416	4.9	0
2015/7/15	17	73	10.55737036	5.5	0
2015/7/16	16.9	63	9.05669945	5	0
2015/7/17	21.3	54	10.06029616	2.1	0
2015/7/18	21.2	75	13.8919563	2.1	0
2015/7/19	21.5	84	15.83142441	2	0
2015/7/20	22.2	74	14.52086161	2.3	0
2015/7/21	22.2	80	15.69822876	2.5	0
2015/7/22	23.6	86	18.28132709	3.2	0
2015/7/23	23.9	86	18.59532538	2.1	0
2015/7/24	23.1	82	16.94170904	2.7	0
2015/7/25	23	84	17.25613708	4.3	0
2015/7/26	23.1	86	17.76813387	2.9	0
2015/7/27	25.1	79	18.27855846	2	0
2015/7/28	25.1	77	17.81581015	2.3	0
2015/7/29	25	79	18.17614461	1.8	0
2015/7/30	25.4	77	18.11818025	1.9	0
2015/7/31	26.4	74	18.41173786	2.7	0
2015/8/1	23.3	78	16.3000979	4.4	0
2015/8/2	23.5	70	14.79578619	2.2	0

2015/8/3	23.9	75	16.21685353	2.2	0
2015/8/4	26.6	71	17.86267396	3.7	0
2015/8/5	27.9	65	17.57037453	3.1	0
2015/8/6	24.7	87	19.68166618	2.2	0
2015/8/7	23.7	79	16.88898949	2.2	0
2015/8/8	23.1	70	14.46243454	3.9	0
2015/8/9	24	70	15.22175844	4.3	0
2015/8/10	25.5	77	18.21994564	3.5	0
2015/8/11	24.1	82	17.9324714	2.1	0
2015/8/12	23.6	80	17.00588567	2.4	0
2015/8/13	23.7	76	16.24763546	2	0
2015/8/14	24	68	14.78685105	3.3	0
2015/8/15	22.8	79	16.0445425	1.8	0
2015/8/16	23	79	16.22898606	1.6	0
2015/8/17	23.5	74	15.64125968	4.2	0
2015/8/18	18.4	87	13.67666806	2.2	0
2015/8/19	19.4	81	13.50745615	4.1	0
2015/8/20	20.5	77	13.69406601	2.6	0
2015/8/21	21.7	73	13.91807752	1.8	0
2015/8/22	23.2	72	14.96073625	2.7	0
2015/8/23	21	70	12.81635208	3.2	0
2015/8/24	20.5	63	11.20423583	2.6	0
2015/8/25	19.6	55	9.280086299	2	0
2015/8/26	19.4	65	10.83931666	6.1	0
2015/8/27	19.9	72	12.36396108	4.4	0
2015/8/28	19.6	65	10.96737472	3.5	0
2015/8/29	19.8	66	11.2674553	4	0
2015/8/30	19.8	71	12.1210504	2.9	0
2015/8/31	21.8	74	14.19034499	1.8	0
2015/9/1	21.8	73	13.99858357	1.9	1
2015/9/2	19.6	82	13.83576503	4	0
2015/9/3	22.5	67	13.37562765	2.3	0
2015/9/4	19.8	79	13.48680256	3.2	0
2015/9/5	18.1	67	10.34682727	3	0
2015/9/6	17.7	67	10.10352952	1.7	0
2015/9/7	19.4	67	11.1728341	2	0
2015/9/8	19.7	64	10.86217046	2.1	0
2015/9/9	19	62	10.09843371	2	0
2015/9/10	20.6	63	11.26967069	7.4	0
2015/9/11	21.9	67	12.92227217	2.8	0
2015/9/12	20.8	74	13.39227296	2.7	0
2015/9/13	15.9	85	11.50526591	1.7	0
2015/9/14	15.7	78	10.43077063	1.4	0
2015/9/15	16.9	67	9.631727986	2.3	0
2015/9/16	18.4	71	11.16141876	2.3	0
2015/9/17	18.8	62	9.979954004	2.9	0
2015/9/18	16.9	76	10.92554219	2.2	0
2015/9/19	16.5	82	11.50821897	1.1	0
2015/9/20	18.4	77	12.10463725	3.7	0
2015/9/21	18.7	75	12.00141001	1.6	0

2015/9/22	19.3	75	12.43358429	2.5	0
2015/9/23	19.3	71	11.77045979	1.6	0
2015/9/24	19	73	11.8900913	3.1	0
2015/9/25	18.8	69	11.106723	4.4	0
2015/9/26	18.1	76	11.73669959	4	0
2015/9/27	17.8	73	11.07408442	3.1	0
2015/9/28	15.3	67	8.744982376	3	0
2015/9/29	12.9	68	7.660140807	4.4	0
2015/9/30	13.4	62	7.203573044	4.8	0
2015/10/1	14.7	69	8.682844066	5.8	0
2015/10/2	15.4	59	7.747717726	6.4	0
2015/10/3	14.2	48	5.858257068	4.2	0
2015/10/4	11.8	57	5.996037544	3.1	0
2015/10/5	11.5	70	7.226532892	1.6	0
2015/10/6	12.3	66	7.162825632	1.7	0
2015/10/7	13.8	66	7.85965044	2.7	0
2015/10/8	11.7	70	7.317632577	9.6	0
2015/10/9	12.4	75	8.190401277	7	0
2015/10/10	14.3	57	6.999444068	3.8	0
2015/10/11	14.5	65	8.080133052	6.3	0
2015/10/12	12.1	60	6.430993015	3.1	0
2015/10/13	8.4	68	5.765577074	3	0
2015/10/14	8.5	58	4.949423131	3.4	0
2015/10/15	10.1	59	5.576292608	2.7	0
2015/10/16	11.2	67	6.787797598	1.3	0
2015/10/17	13.2	70	8.03321847	1.6	0
2015/10/18	14	60	7.233506441	4.7	0
2015/10/19	9.9	46	4.292772714	3.6	0
2015/10/20	9.1	66	5.852989783	3.7	0
2015/10/21	9.2	48	4.284019649	2.8	0
2015/10/22	8.3	50	4.212198296	3.2	0
2015/10/23	10.8	52	5.137145576	2.9	0
2015/10/24	10.6	65	6.340893505	5	0
2015/10/25	4.4	67	4.372658973	10	0
2015/10/26	6.5	51	3.822904772	5.1	0
2015/10/27	9.3	69	6.197740263	4.9	0
2015/10/28	12	57	6.071436467	4.4	0
2015/10/29	7.5	55	4.400035692	2.5	0
2015/10/30	6.3	61	4.513052472	2.5	0
2015/10/31	6.7	51	3.873170653	2.3	0
2015/11/1	8.3	49	4.12795433	1.8	0
2015/11/2	8.7	62	5.359159682	1.7	0
2015/11/3	9.1	67	5.941671446	2.8	0
2015/11/4	11.4	42	4.308811956	3.1	0
2015/11/5	13	57	6.460896903	2.4	0
2015/11/6	8.6	57	4.89544104	4.3	0
2015/11/7	4	50	3.177319079	2.2	0
2015/11/8	4.3	71	4.602967024	1.2	0
2015/11/9	8.5	73	6.229446355	1.7	0
2015/11/10	4.9	89	6.004548485	1.8	0

2015/11/11	5.3	78	5.403516525	2.8	0
2015/11/12	5.3	69	4.780033849	1.5	0
2015/11/13	5.2	69	4.748558056	1.4	0
2015/11/14	9.4	76	6.870203768	5.5	0
2015/11/15	11	92	9.204043771	3.4	0
2015/11/16	10	70	6.57409526	3.3	0
2015/11/17	8.9	74	6.478967481	3	0
2015/11/18	4.8	52	3.485095592	5.1	0
2015/11/19	2.9	53	3.128360581	2.3	0
2015/11/20	4.2	68	4.379198888	1.6	0
2015/11/21	3.8	67	4.201078438	4.3	0
2015/11/22	1.6	43	2.324021391	4.5	0
2015/11/23	1.8	46	2.520257895	2.4	0
2015/11/24	-0.2	87	4.155170549	2.5	0
2015/11/25	-1.1	75	3.364834055	2.2	0
2015/11/26	-1.6	75	3.249214813	1.9	0
2015/11/27	2.8	85	4.983462893	3.1	0
2015/11/28	2.2	81	4.560081566	4	0
2015/11/29	2.3	60	3.400811887	2.4	0
2015/11/30	1.7	73	3.972405219	2.8	0
2015/12/1	0.7	60	3.049133738	5.4	0
2015/12/2	2.1	62	3.466833466	2.7	0
2015/12/3	7	75	5.808306086	5	0
2015/12/4	0.9	65	3.34888494	2.5	0
2015/12/5	-0.5	77	3.601871536	2	0
2015/12/6	-0.9	59	2.684169693	2.7	0
2015/12/7	1.3	68	3.600710391	2.9	0
2015/12/8	1.3	62	3.28300065	1.5	1
2015/12/9	2.8	64	3.752254414	2	0
2015/12/10	6.3	68	5.03094374	2	0
2015/12/11	5	78	5.297384557	3.6	0
2015/12/12	2.5	64	3.676980107	2.6	0
2015/12/13	2.5	67	3.84933855	1.9	0
2015/12/14	2.1	56	3.131333453	1.5	0
2015/12/15	5.4	74	5.16036456	3.8	0
2015/12/16	6	71	5.150586731	3	0
2015/12/17	1	82	4.253806006	4.5	0
2015/12/18	-0.2	78	3.72532532	2.1	0
2015/12/19	-1.2	55	2.450379385	6.2	0
2015/12/20	1.7	58	3.156157572	2.7	0
2015/12/21	2.8	87	5.100720844	1.6	0
2015/12/22	0.5	65	3.258116454	4.7	0
2015/12/23	0	70	3.389814865	2.6	0
2015/12/24	4.2	67	4.314798905	1.3	0
2015/12/25	-1	80	3.614277371	4.6	0
2015/12/26	-4.7	67	2.328856976	2.1	0
2015/12/27	-6.2	59	1.839456976	4.3	0
2015/12/28	-5.4	72	2.379238169	2.6	0
2015/12/29	-4.6	57	1.99558064	3.4	0
2015/12/30	-3.6	73	2.745553649	1.6	0

2015/12/31	-1.6	63	2.729340443	1.9	0
2016/1/1	-1.1	59	2.64700279	3.5	0
2016/1/2	1.6	75	4.053525681	2.6	0
2016/1/3	0.3	52	2.570835318	2.3	0
2016/1/4	-1.7	57	2.452149852	2.3	0
2016/1/5	-3.9	61	2.245597577	2.8	0
2016/1/6	-2.3	66	2.722011684	2.4	0
2016/1/7	-2.7	58	2.32542864	3	0
2016/1/8	-2.5	68	2.76518906	1.7	0
2016/1/9	-3.6	62	2.331840085	2.2	0
2016/1/10	-4.2	69	2.486116934	2.4	0
2016/1/11	-6.7	48	1.442762537	3.4	0
2016/1/12	-6.4	68	2.089303007	2.2	0
2016/1/13	-4.2	60	2.161840812	1.6	0
2016/1/14	-3.7	72	2.688689462	3.1	0
2016/1/15	-6.3	69	2.135578666	1.9	0
2016/1/16	-5	68	2.313028957	1.9	0
2016/1/17	-5.1	73	2.46523038	1.7	0
2016/1/18	-5.9	74	2.358119657	1.9	0
2016/1/19	-1.4	80	3.514707541	3.6	0
2016/1/20	-0.5	60	2.806653145	5.7	0
2016/1/21	-2.1	65	2.718809658	3.7	0
2016/1/22	-2.7	72	2.886739001	3.1	0
2016/1/23	-3.7	59	2.203231642	1.5	0
2016/1/24	-5.7	68	2.198669556	1.6	0
2016/1/25	-6.7	73	2.194201359	1.8	0
2016/1/26	0	66	3.196111159	2.4	1
2016/1/27	-1.7	71	3.054432272	2.9	0
2016/1/28	-3.9	62	2.282410652	3.8	0
2016/1/29	-5	48	1.632726322	4.4	0
2016/1/30	-5.8	66	2.118546126	1.5	0
2016/1/31	-5.6	55	1.791298642	3.7	0
2016/2/1	-5.5	45	1.476281839	3.7	0
2016/2/2	-4.5	58	2.045248423	3.4	0
2016/2/3	-3.5	62	2.348523772	2.6	0
2016/2/4	-0.4	65	3.061710834	2.5	0
2016/2/5	-2.4	66	2.702876874	2.4	0
2016/2/6	-4.9	61	2.089953959	2	0
2016/2/7	-4.7	60	2.085543561	3.2	0
2016/2/8	-3.5	60	2.27276494	3.2	0
2016/2/9	1.8	69	3.780386842	4.5	0
2016/2/10	-1.7	79	3.398593655	7.7	0
2016/2/11	-1.8	59	2.520440733	4.4	0
2016/2/12	0.2	68	3.33875667	3.7	0
2016/2/13	4.8	83	5.562748733	3.6	0
2016/2/14	3.4	87	5.310870845	5.1	0
2016/2/15	-5.7	57	1.843002422	3.9	0
2016/2/16	-5.4	66	2.180968322	1.7	0
2016/2/17	-4.4	67	2.379653703	3	0
2016/2/18	-0.6	76	3.530491285	2.1	0

2016/2/19	2.5	72	4.136602621	2.7	0
2016/2/20	1.5	57	3.059742999	2.4	0
2016/2/21	-0.5	80	3.742204193	4.2	0
2016/2/22	-2.9	59	2.33225134	3.6	0
2016/2/23	-1.9	78	3.308786472	4.2	0
2016/2/24	-6.8	74	2.20800488	4.3	0
2016/2/25	-6.4	60	1.843502653	2.8	0
2016/2/26	-5.6	66	2.149558371	2.3	0
2016/2/27	-4.7	60	2.085543561	2.5	0
2016/2/28	-3.4	49	1.869359469	2.7	0
2016/2/29	0.7	69	3.506503799	6.4	0
2016/3/1	-2	75	3.159238353	7.3	0
2016/3/2	-3.3	63	2.420627935	2.5	0
2016/3/3	-3.4	76	2.899414687	2.2	0
2016/3/4	-2.5	67	2.724524515	2.7	0
2016/3/5	-0.9	54	2.456697685	2.1	0
2016/3/6	3.8	79	4.953510397	8.7	0
2016/3/7	4.7	66	4.394139458	2.6	0
2016/3/8	3.1	59	3.529739703	4.7	0
2016/3/9	-0.6	49	2.276237802	4.2	0
2016/3/10	-2	67	2.822252928	1.4	0
2016/3/11	-2.4	63	2.580018834	2.6	0
2016/3/12	-2	59	2.485267504	2.4	0
2016/3/13	-0.7	64	2.952452759	3.6	0
2016/3/14	2.1	69	3.858250148	2.4	0
2016/3/15	1	49	2.541908467	5.3	0
2016/3/16	5.4	49	3.416998155	3.8	0
2016/3/17	7.9	48	3.940726283	2.6	0
2016/3/18	8.4	58	4.917698092	3.6	0
2016/3/19	5.7	82	5.832413066	4.2	0
2016/3/20	1.1	62	3.238395614	6.1	0
2016/3/21	0.1	62	3.02322095	3.7	0
2016/3/22	2.4	47	2.682072138	2.9	0
2016/3/23	1.8	68	3.725598627	3.7	0
2016/3/24	1	74	3.838800542	2.4	0
2016/3/25	1	78	4.046303274	3.6	0
2016/3/26	3.3	67	4.062610471	4.3	0
2016/3/27	6.5	49	3.672986938	3.1	0
2016/3/28	5.7	54	3.840857385	3.5	0
2016/3/29	6.4	51	3.797985514	3.4	0
2016/3/30	6.6	73	5.507873271	5.8	0
2016/3/31	5.9	62	4.468253668	3.5	0
2016/4/1	7	61	4.72408895	3.1	0
2016/4/2	4.7	61	4.061250105	7.8	0
2016/4/3	7.9	55	4.515415532	4.7	0
2016/4/4	7	43	3.330095489	2.6	0
2016/4/5	8.5	39	3.328060381	4.4	0
2016/4/6	9	34	2.99594638	4.9	0
2016/4/7	8.2	54	4.519965813	4.7	0
2016/4/8	8.8	56	4.871679825	3.1	0

2016/4/9	6.6	36	2.716211476	5.7	0
2016/4/10	4.3	38	2.463559816	4	0
2016/4/11	2.2	70	3.94081123	5.5	0
2016/4/12	6.9	41	3.154597003	4.2	1
2016/4/13	8.1	59	4.906746682	4.5	0
2016/4/14	11	74	7.403252598	3	0
2016/4/15	4.7	73	4.860184552	9.2	0
2016/4/16	8	50	4.131516695	2.8	0
2016/4/17	8.8	75	6.524571194	4.3	0
2016/4/18	6.8	72	5.503791021	5.4	0
2016/4/19	4.4	80	5.221085341	2.2	0
2016/4/20	9.7	53	4.883523076	3.8	0
2016/4/21	13.8	54	6.430623087	3.5	0
2016/4/22	14.1	50	6.065037677	2.6	0
2016/4/23	9.5	70	6.368299716	6.4	0
2016/4/24	9.8	56	5.192875356	2.7	0
2016/4/25	10.3	57	5.455979031	2.8	0
2016/4/26	8.5	64	5.461432421	3.8	0
2016/4/27	8.1	53	4.407755494	3.5	0
2016/4/28	7.6	60	4.83124087	5.1	0
2016/4/29	4.8	79	5.294664456	5.3	0
2016/4/30	4.3	63	4.084322852	7.1	0
2016/5/1	6.3	59	4.365083539	3	0
2016/5/2	9.9	53	4.946020736	3.5	0
2016/5/3	16.4	60	8.370114939	7	0
2016/5/4	13.6	78	9.175015139	5.6	0
2016/5/5	12	68	7.243117188	3.4	0
2016/5/6	11.5	72	7.43300526	2.8	0
2016/5/7	12.5	67	7.362409327	5.7	0
2016/5/8	15.2	34	4.410859807	6.8	0
2016/5/9	14.1	42	5.094631648	1.8	0
2016/5/10	15.8	42	5.650672351	2.9	0
2016/5/11	11.4	81	8.30985163	2.4	0
2016/5/12	10.4	79	7.609806911	4.7	0
2016/5/13	15.6	54	7.177684481	4.5	0
2016/5/14	14.5	45	5.593938267	2.8	0
2016/5/15	14.7	26	3.271796315	7.4	0
2016/5/16	12.6	69	7.629450451	12.5	0
2016/5/17	16.5	54	7.578583224	3.7	0
2016/5/18	16	56	7.625889722	2.5	0
2016/5/19	17.6	51	7.645039519	3.4	0
2016/5/20	19.1	47	7.700516055	1.4	0
2016/5/21	19.9	43	7.384032311	1.8	0
2016/5/22	18.6	61	9.70360106	2.5	0
2016/5/23	17.2	64	9.367280306	2.7	0
2016/5/24	16.9	55	7.906642377	5.9	0
2016/5/25	16.9	71	10.20675652	7.4	0
2016/5/26	19.8	56	9.560265106	3.6	0
2016/5/27	17.3	47	6.920357969	3.9	0
2016/5/28	15.5	55	7.266410955	3.2	0

2016/5/29	14.2	67	8.17715049	3	0
2016/5/30	16.2	57	7.856343334	6	0
2016/5/31	15.1	77	9.92871639	5	0
2016/6/1	11.7	80	8.363008659	4.8	0
2016/6/2	10.8	64	6.322640709	6.8	0
2016/6/3	11.3	78	7.952009683	1.8	1
2016/6/4	13.4	83	9.643492947	3.3	0
2016/6/5	12.2	66	7.118339058	5.6	0
2016/6/6	14.7	58	7.298622548	2	0
2016/6/7	18.6	52	8.271922215	2	0
2016/6/8	17.2	73	10.6845541	2.9	0
2016/6/9	16.6	85	12.00121338	2	0
2016/6/10	14.8	82	10.38191292	5.6	0
2016/6/11	15.2	75	9.729837809	3.6	0
2016/6/12	16.1	70	9.590100405	8.1	0
2016/6/13	16.5	80	11.2275307	8.6	0
2016/6/14	15.9	77	10.42241736	6.1	0
2016/6/15	16.8	70	10.00280587	6.3	0
2016/6/16	15.2	79	10.24876249	6.4	0
2016/6/17	16.1	88	12.05612622	4	0
2016/6/18	14	86	10.3680259	4.3	0
2016/6/19	16.9	73	10.49427079	4	0
2016/6/20	17.7	85	12.81791058	5.3	0
2016/6/21	20.3	72	12.65639837	3.7	0
2016/6/22	19.8	73	12.46248844	2.6	0
2016/6/23	19.1	65	10.64964986	3.7	0
2016/6/24	17.9	64	9.766737352	4.9	0
2016/6/25	15.8	82	11.03226507	7	0
2016/6/26	15.8	80	10.76318543	4.9	0
2016/6/27	16.5	71	9.964433498	3.4	0
2016/6/28	19.5	65	10.90318343	2	0
2016/6/29	21.1	63	11.60181199	2	0
2016/6/30	21.6	65	12.32148082	3.8	0
2016/7/1	22.1	66	12.87679348	2.4	0
2016/7/2	20.4	82	14.49854874	6	0
2016/7/3	21.2	71	13.15105197	4.3	0
2016/7/4	16.5	75	10.52581003	6.1	0
2016/7/5	17.7	70	10.55592636	3.1	0
2016/7/6	16.8	69	9.859908642	5.4	0
2016/7/7	18.6	68	10.81712905	2.3	0
2016/7/8	19.5	71	11.90963114	3.1	1
2016/7/9	20.9	73	13.28826713	2.3	0
2016/7/10	18.4	89	13.99107423	3.3	0
2016/7/11	17.8	79	11.98428314	5.2	0
2016/7/12	19.6	74	12.48593429	3.1	0
2016/7/13	21.8	70	13.42329932	3.5	0
2016/7/14	21.2	65	12.03969546	4.7	0
2016/7/15	21.6	69	13.0797258	2.3	0
2016/7/16	21.9	73	14.07949057	2	0
2016/7/17	20.1	76	13.20441155	4.6	0

2016/7/18	22	72	13.9668166	2.9	0
2016/7/19	19.9	87	14.9397863	2.1	1
2016/7/20	20.8	77	13.93520295	2.2	0
2016/7/21	21.2	73	13.52150413	2.2	0
2016/7/22	20.7	73	13.13471022	2.9	0
2016/7/23	20.7	69	12.41500007	4.3	0
2016/7/24	20.6	68	12.164089	5.1	0
2016/7/25	21.1	71	13.07505795	4.5	0
2016/7/26	22.1	71	13.85230814	5.4	0
2016/7/27	20.6	86	15.38399491	7.8	0
2016/7/28	22.8	87	17.6693063	4	0
2016/7/29	23.2	88	18.28534431	4	0
2016/7/30	24.6	78	17.54645275	5	0
2016/7/31	25.4	76	17.8828792	3.8	0
2016/8/1	26.5	74	18.51434098	4.3	0
2016/8/2	25.1	82	18.97268094	2.8	1
2016/8/3	25	80	18.40622239	2	0
2016/8/4	24.9	74	16.93028626	1.9	0
2016/8/5	23.5	68	14.37304944	3	0
2016/8/6	23.2	63	13.09064422	2.3	0
2016/8/7	25.9	67	16.21220384	4.3	0
2016/8/8	26.5	73	18.26414718	5.3	0
2016/8/9	23	70	14.38011423	6.4	0
2016/8/10	22.3	68	13.4203692	4.7	0
2016/8/11	23.4	66	13.87115817	2.1	0
2016/8/12	24.1	69	15.08951861	2.3	0
2016/8/13	24.2	60	13.19578133	4.6	0
2016/8/14	24.4	64	14.23551364	5.1	0
2016/8/15	24.8	72	16.38026998	3.7	0
2016/8/16	24.2	79	17.37444541	4	0
2016/8/17	22.2	92	18.05296308	3.3	0
2016/8/18	24.5	75	16.77668611	2.3	0
2016/8/19	24.3	78	17.25178694	2.5	0
2016/8/20	23.4	89	18.70504662	2.1	0
2016/8/21	24.7	86	19.45544014	2.6	0
2016/8/22	23.9	90	19.46022423	2	0
2016/8/23	22.7	83	16.76074643	1.9	0
2016/8/24	23.1	69	14.25582834	1.8	1
2016/8/25	23.7	73	15.60628143	5.2	0
2016/8/26	20.5	72	12.80484095	3.5	1
2016/8/27	20.3	59	10.37121533	2.9	0
2016/8/28	20.5	61	10.8485458	2.3	0
2016/8/29	25.8	64	15.40005821	2.8	0
2016/8/30	27.1	68	17.58841382	4.4	0
2016/8/31	24.1	77	16.83902802	6	0
2016/9/1	25.8	67	16.12193594	3.6	0
2016/9/2	24.2	76	16.71465635	2	0
2016/9/3	23.5	74	15.64125968	4.4	0
2016/9/4	22.8	71	14.41977871	4.5	0
2016/9/5	23.7	74	15.82006611	4.7	0

2016/9/6	23.3	87	18.18087843	3	0
2016/9/7	22.6	76	15.25955598	2.7	0
2016/9/8	18.5	77	12.1765383	2.7	0
2016/9/9	17.9	83	12.6662375	2.1	0
2016/9/10	18.6	85	13.52141131	3	0
2016/9/11	18.3	71	11.09545879	3.3	0
2016/9/12	18.2	65	10.09773698	2.1	0
2016/9/13	18.9	70	11.33440333	4.3	0
2016/9/14	19.3	71	11.77045979	3.9	0
2016/9/15	19.8	79	13.48680256	1.9	0
2016/9/16	21	68	12.45017059	2.9	0
2016/9/17	18.1	75	11.58226933	3.7	0
2016/9/18	15.3	74	9.658637251	2	0
2016/9/19	14.9	71	9.04420354	2	0
2016/9/20	16.1	60	8.220086061	1.7	0
2016/9/21	16.3	57	7.903852121	2	0
2016/9/22	16.4	70	9.765134095	4.3	0
2016/9/23	16.3	88	12.20243836	2.4	0
2016/9/24	18.6	78	12.40788332	1.8	0
2016/9/25	20.9	66	12.01404974	3.3	0
2016/9/26	20.3	63	11.07434857	2.3	0
2016/9/27	19.9	59	10.13157922	3.4	0
2016/9/28	19	82	13.35599297	3.3	0
2016/9/29	16.1	66	9.042094668	3.4	0
2016/9/30	16.6	58	8.189063247	2.3	0
2016/10/1	17.6	61	9.144066875	1.9	0
2016/10/2	18.4	63	9.903794115	1.8	0
2016/10/3	18.1	79	12.19999036	3.3	0
2016/10/4	15.5	49	6.473711578	6.5	0
2016/10/5	13.3	60	6.928293245	3.1	0
2016/10/6	12.7	63	7.009410545	5.6	0
2016/10/7	10.9	45	4.473715265	5	0
2016/10/8	13.3	74	8.544895002	3.6	0
2016/10/9	12.9	55	6.195702123	5.1	0
2016/10/10	9.1	53	4.700128159	2.8	0
2016/10/11	11.3	59	6.014981683	2.8	0
2016/10/12	10.2	48	4.565497131	3.9	0
2016/10/13	10.7	49	4.810331826	2.4	0
2016/10/14	10.6	46	4.487401557	4	0
2016/10/15	11.3	56	5.709135157	1.8	0
2016/10/16	15.2	70	9.081181955	1.7	0
2016/10/17	16.1	74	10.13810614	3.5	0
2016/10/18	12.6	70	7.740022197	3.8	0
2016/10/19	11.5	67	6.916824339	1.7	0
2016/10/20	7.8	71	5.791439816	6.5	0
2016/10/21	5.4	56	3.905140748	5.8	0
2016/10/22	7.3	65	5.133007388	2	0
2016/10/23	6.5	55	4.12274044	4.6	1
2016/10/24	6.5	38	2.84843885	6.7	0
2016/10/25	8.4	56	4.748122296	3.1	0

2016/10/26	10.5	58	5.622389911	4.7	0
2016/10/27	6.6	43	3.244363708	4.5	0
2016/10/28	8.2	46	3.850341248	2.9	0
2016/10/29	4.3	48	3.11186503	6.9	0
2016/10/30	3	51	3.030660105	2.9	0
2016/10/31	4.3	69	4.473305981	4.2	0
2016/11/1	2.7	52	3.028199616	3.3	0
2016/11/2	2.9	52	3.069334909	2.1	0
2016/11/3	2.4	80	4.565229171	1.7	0
2016/11/4	2.8	51	2.990077736	3.6	0
2016/11/5	1.8	87	4.766574714	3.3	0
2016/11/6	0.9	67	3.451927553	5.6	0
2016/11/7	1.8	57	3.122928261	2.9	0
2016/11/8	5.5	75	5.264707855	4.9	0
2016/11/9	-0.1	67	3.222180595	4.2	0
2016/11/10	0.5	54	2.7067429	4	0
2016/11/11	1.7	63	3.428240121	2.6	0
2016/11/12	5	81	5.501130116	2	0
2016/11/13	6.9	76	5.847545663	4	0
2016/11/14	6.7	65	4.936393969	4.4	0
2016/11/15	5.8	77	5.512926628	4.7	0
2016/11/16	0.7	57	2.896677051	4.6	0
2016/11/17	0.5	55	2.756867769	2.7	0
2016/11/18	5.1	69	4.717263023	3.4	0
2016/11/19	9.4	81	7.322190858	2.5	0
2016/11/20	6.2	68	4.998094626	4.7	0
2016/11/21	1	51	2.645659833	3.2	0
2016/11/22	0.1	74	3.608360489	3.3	0
2016/11/23	-5.2	52	1.743402666	5.5	0
2016/11/24	-3.5	49	1.856091368	2.1	0
2016/11/25	-2	58	2.443144326	2.7	0
2016/11/26	0.6	50	2.523541969	3.3	0
2016/11/27	3.6	78	4.825761107	3.5	0
2016/11/28	1.8	69	3.780386842	4.4	0
2016/11/29	-0.8	66	3.023609643	4.3	0
2016/11/30	-0.5	59	2.759875592	3.3	1
2016/12/1	5.8	69	4.940155031	5.1	0
2016/12/2	1.1	64	3.342859989	4.6	0
2016/12/3	3.6	45	2.784092947	3.1	0
2016/12/4	7.1	67	5.222625079	4	0
2016/12/5	6.3	74	5.47485054	3.4	0
2016/12/6	-2.1	77	3.220743749	5.4	0
2016/12/7	-5	51	1.734771718	3.7	0
2016/12/8	-2.5	66	2.68385997	1.5	0
2016/12/9	-0.3	86	4.079058811	1.9	0
2016/12/10	-2.6	82	3.311011352	4.1	0
2016/12/11	-4.6	71	2.485723253	5.5	0
2016/12/12	-4.1	72	2.612868669	1.9	0
2016/12/13	0.2	74	3.633352847	3.4	0
2016/12/14	-2	76	3.201361531	1.5	0

2016/12/15	-4.5	71	2.503666173	2.2	0
2016/12/16	-5.3	74	2.463105459	1.7	0
2016/12/17	-3.6	62	2.331840085	3.4	0
2016/12/18	1	64	3.320043712	2.8	0
2016/12/19	1	61	3.164416663	2.4	0
2016/12/20	-0.1	68	3.270272842	2.4	1
2016/12/21	2.7	65	3.78524952	3.1	0
2016/12/22	0.3	85	4.202326962	1.6	0
2016/12/23	-1.4	88	3.866178295	3.1	0
2016/12/24	-4.4	60	2.131033167	2.8	0
2016/12/25	-2.3	62	2.557041279	2.7	0
2016/12/26	1.3	77	4.077275001	3.8	0
2016/12/27	-2.5	70	2.84651815	3.2	0
2016/12/28	-6.4	64	1.96640283	2.1	0
2016/12/29	-3.6	66	2.482281381	4.6	0
2016/12/30	-2.8	58	2.309024418	2.7	0
2016/12/31	-2.6	64	2.584203982	3.6	0

Table S2. Background demographic characteristics, date of sampling and admitted ward of cases.

Date of sampling	Bacterium	Age	Sex	Ward
2011/1/14	Bacillus cereus	45	F	Gynecology
2011/6/14	Bacillus subtilis	80	F	Hematology
2011/6/27	Bacillus subtilis	69	M	Respiratory medicine
2012/3/15	Bacillus cereus	52	F	Mammal surgery
2012/3/27	Bacillus cereus	62	F	Gynecology
2012/8/6	Bacillus subtilis	47	F	Urology
2012/8/11	Bacillus cereus	82	F	Hematology
2012/11/5	Bacillus cereus	84	M	Gastroenterology
2012/12/23	Bacillus cereus	71	M	Respiratory medicine
2013/5/6	Bacillus species	79	F	Urology
2013/7/2	Bacillus cereus	72	M	Urology
2013/8/23	Bacillus cereus	65	M	Urology
2013/8/31	Bacillus cereus	14	F	Ongology
2013/9/21	Bacillus cereus	57	F	Gynecology
2013/10/3	Bacillus cereus	60	M	Urology
2013/10/15	Bacillus cereus	90	M	Urology
2013/10/24	Bacillus cereus	72	M	Gastroenterology
2014/2/7	Bacillus megaterium	69	M	Urology
2014/2/28	Bacillus cereus	64	M	Gastroenterology
2014/4/13	Bacillus cereus	58	M	Head and neck surgery
2014/4/21	Bacillus cereus	53	F	Hematology
2014/7/25	Bacillus cereus	55	F	Gynecology
2014/7/28	Bacillus cereus	59	M	Urology
2014/8/1	Bacillus cereus	78	F	Hematology
2014/8/5	Bacillus cereus	61	F	Gynecology
2014/9/2	Bacillus cereus	48	F	Gastroenterology
2014/10/8	Bacillus cereus	81	M	Urology
2014/11/13	Bacillus cereus	65	M	Respiratory medicine

2014/11/24	Bacillus subtilis	53	F	Gastroenterology
2014/12/20	Bacillus cereus	22	M	Ongology
2015/4/22	Bacillus cereus	78	M	Respiratory medicine
2015/4/28	Bacillus cereus	50	F	Gynecology
2015/5/28	Bacillus species	69	M	Hematology
2015/6/5	Bacillus species	56	F	Mammal surgery
2015/6/7	Bacillus cereus	54	F	Mammal surgery
2015/6/12	Bacillus cereus	72	F	Respiratory medicine
2015/6/24	Bacillus megaterium	64	M	Hematology
2015/6/28	Bacillus cereus	59	M	Ongology
2015/9/1	Bacillus cereus	69	M	Respiratory medicine
2015/12/8	Bacillus cereus	72	M	Urology
2016/1/26	Bacillus cereus	77	F	Respiratory medicine
2016/4/12	Bacillus cereus	62	M	Gastroenterology
2016/6/3	Bacillus species	72	F	Respiratory medicine
2016/6/3	Bacillus cereus	63	F	Gastroenterology
2016/7/8	Bacillus cereus	67	F	Hematology
2016/7/19	Bacillus cereus	47	F	Mammal surgery
2016/8/2	Bacillus cereus	75	F	Gastroenterology
2016/8/24	Bacillus cereus	68	M	Ongology
2016/8/26	Bacillus cereus	53	F	Gastroenterology
2016/10/23	Bacillus cereus	73	F	Mammal surgery
2016/11/30	Bacillus cereus	71	F	Gynecology
2016/12/20	Bacillus cereus	31	F	Gastroenterology
